# Magnetic-field-guided catalytic effect mitigates Li_2_S passivation of lithium–sulfur batteries

**DOI:** 10.1093/nsr/nwag039

**Published:** 2026-01-19

**Authors:** Lang Liao, Ruijin Meng, Chen Zhou, Shusheng Li, Wei Xu, Xiaoning Li, Kai Zhang, Lu Chen, Fangyi Shi, Duanzijing Liu, Hongying Hou, Chi Zhang, Jinhu Yang

**Affiliations:** School of Chemical Science and Engineering, State Key Laboratory of Cardiovascular Diseases, Shanghai East Hospital, Tongji University, Shanghai 200092, China; Faculty of Material Science and Engineering, Kunming University of Science and Technology, Southwest United Graduate School, Kunming 650092, China; School of Chemical Science and Engineering, State Key Laboratory of Cardiovascular Diseases, Shanghai East Hospital, Tongji University, Shanghai 200092, China; School of Chemical Science and Engineering, State Key Laboratory of Cardiovascular Diseases, Shanghai East Hospital, Tongji University, Shanghai 200092, China; School of Chemical Science and Engineering, State Key Laboratory of Cardiovascular Diseases, Shanghai East Hospital, Tongji University, Shanghai 200092, China; Department of Optometry, Shanghai Eye Diseases Prevention & Treatment Center/Shanghai Eye Hospital, School of Medicine, Tongji University; National Clinical Research Center for Eye Diseases; Shanghai Engineering Research Center of Precise Diagnosis and Treatment of Eye Diseases, Shanghai 200092, China; Centre for Atomaterials and Nanomanufacturing (CAN), School of Science, RMIT University, Melbourne, VIC 3000, Australia; Frontiers Science Center for New Organic Matter, State Key Laboratory of Advanced Chemical Power Sources, Key Laboratory of Advanced Energy Materials Chemistry (Ministry of Education), Collaborative Innovation Center of Chemical Science and Engineering (Tianjin), College of Chemistry, Nankai University, Tianjin 300071, China; School of Chemical Science and Engineering, State Key Laboratory of Cardiovascular Diseases, Shanghai East Hospital, Tongji University, Shanghai 200092, China; Department of Applied Physics, the Hong Kong Polytechnic University, Hong Kong 999077, China; Department of Applied Physics, the Hong Kong Polytechnic University, Hong Kong 999077, China; Faculty of Material Science and Engineering, Kunming University of Science and Technology, Southwest United Graduate School, Kunming 650092, China; School of Chemical Science and Engineering, State Key Laboratory of Cardiovascular Diseases, Shanghai East Hospital, Tongji University, Shanghai 200092, China; School of Chemical Science and Engineering, State Key Laboratory of Cardiovascular Diseases, Shanghai East Hospital, Tongji University, Shanghai 200092, China; School of Materials and Chemical Engineering, Fuyang University of Technology, Fuyang 236115, China

**Keywords:** magnetic field, Li_2_S deposition/dissociation, d–p orbital hybridization, Li–S battery, shuttle effect

## Abstract

Polysulfides shuttle and Li_2_S passivation are considered key problems of lithium–sulfur (Li–S) batteries, seriously hindering sulfur recycling for practical applications. However, the prevailing strategies for introducing electrocatalysts focus mainly on the polysulfides-shuttle effect, whereas Li_2_S passivation involving solid conversions with high energy barriers has been rarely studied and remains challenging. Herein, we propose that applying a magnetic field (MF) to weak ferromagnetism *α*-Fe_2_O_3_ shows an enhanced effect, not only suppressing the polysulfides shuttle, but also alleviating Li_2_S passivation by synchronously catalysing polysulfides conversion and Li_2_S deposition/dissociation. Experimental and theoretical studies reveal that the MF can promote the consistent alignment of magnetic-domain orientations in *α*-Fe_2_O_3_ and enhance the electron spin polarization of the Fe 3d orbital, which shifts the center of the Fe d-band towards the Fermi level and increases the hybridization degree between Fe 3d and S 3p orbitals in *α*-Fe_2_O_3_–Li_2_S_6_ or *α*-Fe_2_O_3_–Li_2_S, thus enhancing the adsorption and catalysis of polysulfides and solid products. As a result, after using a 400-mT MF, the *α*-Fe_2_O_3_/S cathode shows outstanding cycling stability and excellent rate capability. The research demonstrates that MF-guided electrocatalysis represents an effective solution to the challenging problems of Li–S batteries.

## Teaser

Magnetic field-guided catalytic effect over catalyst promotes not only polysulfide conversion but also Li2S deposition/dissociation via forming electroactive loose Li2S, synchronously suppressing polysulfide shuttle and Li2S passivation for high-performance Li-S batteries.

## INTRODUCTION

Lithium–sulfur (Li–S) batteries have been regarded as one of the next-generation power systems because of their high energy density, low cost and environmental compatibility [1]. However, many thorny challenges limit their practical application, such as the inevitable ‘shuttle effect’ of soluble lithium polysulfides (LiPSs) intermediates and the sluggish reaction kinetics of sulfide conversion [2–6]. The weak interaction between the non-polar carbonaceous matrix [7–11] and polysulfides displays a limited effect on suppressing the shuttle effect and accelerating sulfide conversion over long-term cycling. Oxides, nitrides, sulfides, selenides, metal–organic frameworks, MXene and metal–N–C materials of group 3d transition metals are known to have strong adsorption and catalytic abilities for LiPSs [12–20]. In order to further improve the catalytic performance of catalysts, coordination modulation [21,22], defect control [18], heterojunctions [23], element doping [24,25] and applying external fields have been investigated.

It is noted that the whole electrochemical processes of Li–S batteries include conversion reactions from S_8_ to long-chain LiPSs (S_8_→Li_2_S_4_) and subsequently to short-chain Li_2_S_2_ and Li_2_S (Li_2_S_4_→Li_2_S_2_→Li_2_S) during the discharging process, followed by delithiation conversion reactions (Li_2_S→S_8_) during the charging process [1,3]. Relative to LiPS conversions (S_8_→Li_2_S_4_) at solid–liquid-phase interfaces, the short-chain sulfides conversions that undergo solid–solid phase reactions (Li_2_S_2_→Li_2_S) possess higher energy barriers [20], leading to slower conversion kinetics and depositing rates of Li_2_S,

which are prone to forming a Li_2_S passivation film on the electrodes. It is noteworthy that the dissociation of Li_2_S from the passivation film has a high energy barrier during the charging process [12], resulting in low sulfur utilization and thereby a poor cycling performance. However, unlike the polysulfides shuttle that can be mitigated through the intrinsic adsorption/catalytic effect of electrocatalysts, the issues for Li_2_S passivation films are much more challenging and have scarcely been investigated, as they require the regulation of Li_2_S-conversion dynamics and deposition/dissociation behaviors in the solid phase. Therefore, an effective strategy for alleviating Li_2_S passivation is highly desired for the development of Li–S batteries, especially with the simultaneous suppression of polysulfides shuttle through a simple and feasible method.

Recently, applying a magnetic field (MF) to transition-metal catalysts has emerged as a simple and effective approach in various electrochemical systems, including oxygen evolution reactions [26], oxygen reduction reactions [27], hydrogen evolution reactions [17], lithium-ion batteries, Li–O_2_ batteries and Li–S batteries. In lithium-ion batteries, research has focused on studying the inhibition of lithium dendritic growth on the Li anode. Li^+^ ions in the electrolyte are subjected to a Lorentz force that is perpendicular to the electric and magnetic fields. The Lorentz force will change the moving direction of Li^+^ ions, causing spiral motion of the charge and inducing convection in the electrolyte, which can effectively promote the transfer and uniform distribution of Li^+^ ions on the anode [28,29]. In addition, the MF promotes the directional alignment of magnetic material, creating a fast channel for Li^+^-ions migration [30]. In Li–O_2_ batteries, with the introduction of an external MF, the Lorentz force acts oppositely on the photogenerated electrons and holes, suppressing the recombination of charge carriers [31]. In Li–S batteries, an MF was used to investigate the catalyst-assisted trapping of LiPSs to suppress the shuttle effect and enhance the overall battery performance [32–35].

However, the success of previous reports has been limited to liquid-phase- or liquid/solid-interface-involved systems, in which ions or LiPSs under an MF can diffuse freely with low energy barriers. It is noted that Li_2_S passivation is associated with complicated solid-phase-conversion reactions with sluggish kinetics and high energy barriers, and remains a great challenge. Given that MF can regulate the adsorption and catalytic behavior of catalysts and polysulfides, investigating the effects of the MF on the deposition state of Li_2_S on catalyst surfaces and the electronic interactions between catalysts and Li_2_S represents an innovative research direction. To date, the use of an MF to promote Li_2_S deposition/dissociation has not been thoroughly investigated.

In this work, an MF is utilized as a simple and effective regulation means, combined with magnetic *α*-Fe_2_O_3_ as a catalyst [36], to address the key challenging issues of Li_2_S passivation in Li–S batteries. The result demonstrates that MF-guided catalysis promotes not only polysulfides conversion, but also Li_2_S deposition/dissociation through the formation of electroactive loose films, synchronously realizing shuttle-effect suppression and Li_2_S anti-passivation for high-performance Li–S batteries. The mechanism stems from the fact that the MF can enhance the electron spin polarization of the Fe 3d electrons in *α*-Fe_2_O_3_, shifting the center of the Fe d-band towards the Fermi level and increasing the hybridization between Fe 3d and S 3p orbitals in *α*-Fe_2_O_3_–Li_2_S_6_ or *α*-Fe_2_O_3_–Li_2_S, which reinforces the adsorption and catalytic capability of *α*-Fe_2_O_3_ towards sulfur species. This work not only demonstrates a low-cost and effective strategy to promote the catalysis of Li_2_S deposition/dissociation and LiPSs, but also enriches the application of the MF effect in electrocatalysis and energy-storage fields. The additional mass burden and energy consumption introduced by MF-generating devices can be regarded as acceptable in remote and open operating environments, and in the context of large-scale clean-energy conversion and storage. Therefore, the findings of this study demonstrate significant potential for practical applications in such scenarios.

## RESULTS AND DISCUSSION

### Material characterizations and catalytic mechanism under an MF

**Figure 1.**
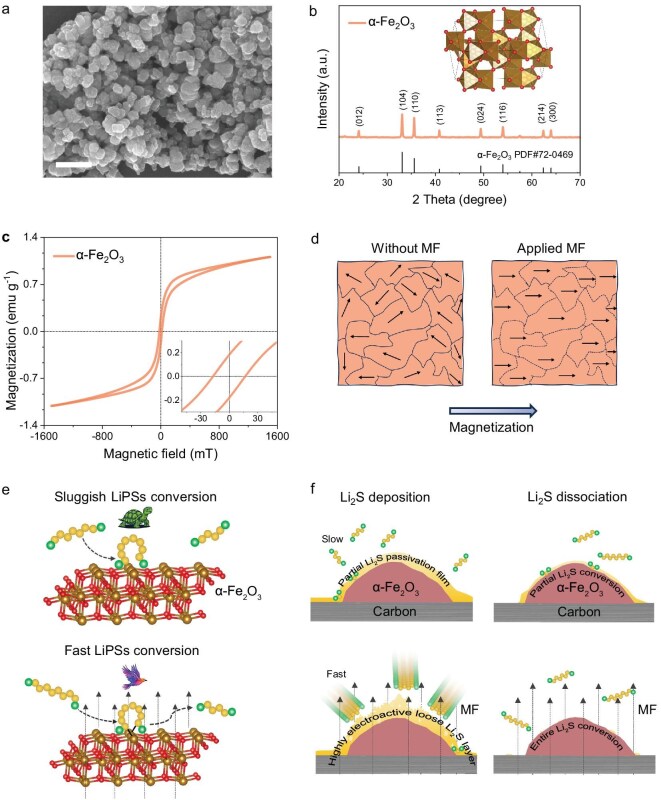
(a) Scanning electron microscopy image. Scale bar is 500 nm. (b) X-ray diffraction pattern and the crystal structure of the *α*-Fe_2_O_3_ particles. (c) Magnetic hysteresis loop of *α*-Fe_2_O_3_; the inset shows a magnified view. (d–f) Schematic diagrams of (d) the magnetic-domain orientations of *α*-Fe_2_O_3_, (e) the conversion of LiPSs and (f) Li_2_S deposition/dissociation with and without MFs.

Figure 1a shows a scanning electron microscopy (SEM) image of *α*-Fe_2_O_3_ in which the *α*-Fe_2_O_3_ particles exhibit an irregular polyhedral shape and have an average diameter of ∼100 nm. The corresponding X-ray diffraction (XRD) pattern in Fig. 1b shows the characteristic peaks of *α*-Fe_2_O_3_ particles with rhombohedral symmetry belonging to the $R\bar{3}c$ space group (PDF#72-0469). The magnetic property of *α*-Fe_2_O_3_ particles was characterized by using a vibrating sample magnetometer (VSM), as shown in Fig. 1c. The Morin transition of *α*-Fe_2_O_3_ particles occurs at ∼263 K (*T*_M_ ≈ 263 K), above which the material becomes weakly ferromagnetic and below which it behaves as an antiferromagnet [37]. Therefore, at room temperature (298 K), *α*-Fe_2_O_3_ particles exhibit weak ferromagnetism with a saturation magnetization of 1.11 emu g^−1^ at 1500 mT, and a coercivity of 16.9 mT (Fig. 1c). When the applied MF is greater than the coercivity of the material, the alignment of the magnetic domain is oriented along the direction of the MF, as shown in Fig. 1d. The consistent alignment of the magnetic-domain orientations facilitates the migration of spin-selective electrons from the catalyst towards the reactants [38]. In this work, MF-guided catalysis using *α*-Fe_2_O_3_ catalyst not only promotes polysulfides conversion, but also facilitates Li_2_S deposition and dissociation, resulting in the formation of a highly electroactive, loosely structured Li_2_S film. This structure is also beneficial for fast and complete Li_2_S dissociation, as shown in Fig. 1e and f. This process simultaneously suppresses the shuttle effect and mitigates Li_2_S passivation, enabling high-performance Li–S batteries.

### Regulation of Li_2_S deposition/dissociation under an MF

**Figure 2.**
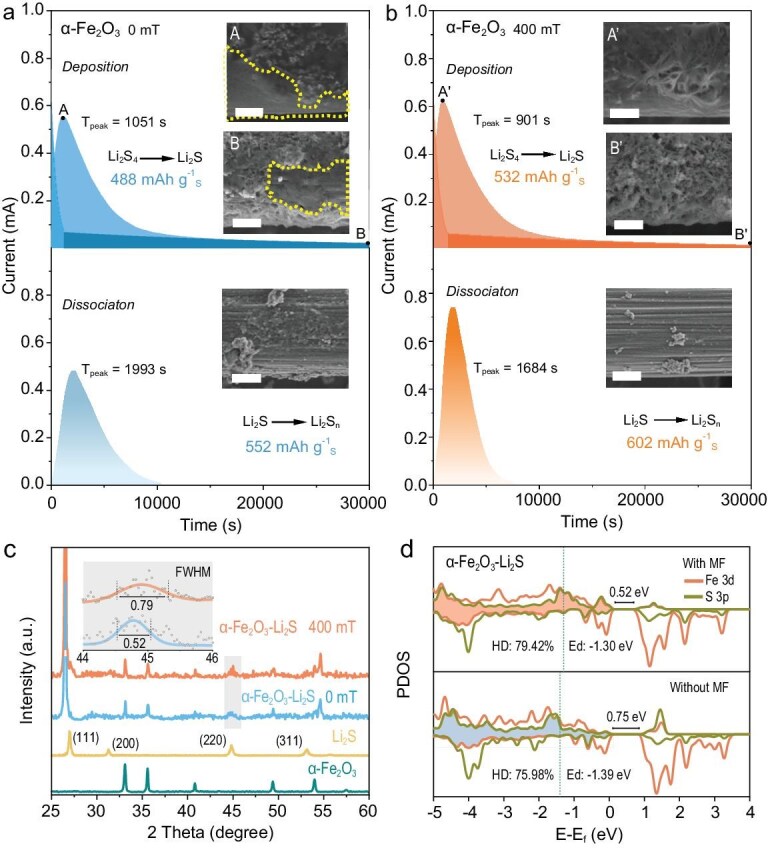
Li_2_S-deposition and -dissociation curves of *α*-Fe_2_O_3_ with MFs of (a) 0 and (b) 400 mT. The insets show SEM images of the carbon nanofibers after Li_2_S deposition and dissociation. Scale bars are 2 μm. (c) XRD patterns of *α*-Fe_2_O_3_–Li_2_S 0 mT and *α*-Fe_2_O_3_–Li_2_S 400 mT, with commercial Li_2_S and *α*-Fe_2_O_3_ powders as references. The inset shows the (220) planes of *α*-Fe_2_O_3_–Li_2_S 0 mT and *α*-Fe_2_O_3_–Li_2_S 400 mT. (d) Projected density of states of Fe 3d and S 3p orbitals of *α*-Fe_2_O_3_–Li_2_S with and without MFs.

Li_2_S deposition and dissociation experiments on *α*-Fe_2_O_3_/carbon paper (CP) were performed. The Li_2_S_4_→Li_2_S reaction contributes three-quarters of the theoretical capacity, while it is also the rate-determining step owing to sluggish kinetics. It thus governs the overall sulfur utilization in the electrode and determines the energy/power density of the Li–S battery. To investigate the effect of the MF on *α*-Fe_2_O_3_-catalysed Li_2_S conversion and deposition/dissociation, experiments were conducted under MFs of 0 and 400 mT, as shown in Fig. 2a and b. After a 400-mT MF was applied, the Li_2_S-deposition capacity catalysed by *α*-Fe_2_O_3_ exhibited an obvious increase from 488 to 532 mAh g^−1^. In addition, the deposition morphology of the Li_2_S on the *α*-Fe_2_O_3_/CP was observed by using SEM. A carbon nanofiber on the surface of the original CP shows a clean surface (Fig. S1). In contrast, the SEM images at points A and B and the energy-dispersive X-ray spectroscopy (EDS) elemental mappings at point B of the Li_2_S deposition with an MF of 0 mT show partially smooth passivation films, as shown by the dashed curves in Fig. 2a and Fig. S2. The passivation film is highly insulating, which reduces the amount and slows the rate of subsequent Li_2_S deposition [39], thereby resulting in a denser passivation layer. However, the Li_2_S product at point A' of the deposition curve under a 400-mT MF exhibits a loose and network-like morphology (Fig. 2b). The loosely stacked Li_2_S sheets serve as nucleation sites for subsequent Li_2_S deposition with a 400-mT MF, resulting in a thicker Li_2_S film with a fluffier and looser morphology (at the end point B'), which is different from the partly dense film at point B with a 0-mT MF, as illustrated in Fig. 2b and a, respectively.

To further investigate the dynamic effects of the MF on Li_2_S nucleation and growth, we compared the Li_2_S-nucleation modes of *α*-Fe_2_O_3_ under an MF of 0 mT/400 mT with theoretical models from Scharifker–Hills. The nucleation rates of 2D progressive nucleation (2DP) and 2D instantaneous nucleation (2DI) are controlled by the crystal phase, whereas the nucleation rates of 3D progressive nucleation (3DP) and 3D instantaneous nucleation (3DI) are primarily determined by ion diffusion [40]. For *α*-Fe_2_O_3_/CP 400 mT, the nucleation of Li_2_S tends to follow the 3DI mode, as depicted in Fig. S3, which results in a faster deposition rate and an earlier attainment of the peak current (A', *T*_peak_ = 901 s; A, *T*_peak_ = 1051s), as shown in Fig. 2a and b. This suggests that a faster nucleation rate allows more achievable Li_2_S deposition (higher capacity) if 3D growth directions are allowed/activated [41]. To better understand the structure of Li_2_S nucleation, we characterized Li_2_S deposition on *α*-Fe_2_O_3_/CP with an MF of 0 mT/400 mT (*α*-Fe_2_O_3_–Li_2_S 0 mT and *α*-Fe_2_O_3_–Li_2_S 400 mT) by using XRD. The XRD measurements (Fig. 2c and Fig. S4) show that the full width at the half maximum (FWHM) of the Li_2_S (220) plane centered at 44.7 for *α*-Fe_2_O_3_–Li_2_S 400 mT is larger than that for *α*-Fe_2_O_3_–Li_2_S 0 mT. This suggests that the Li_2_S in *α*-Fe_2_O_3_–Li_2_S 400 mT possesses a reduced crystallinity with a looser morphology. The oxidation reaction of Li_2_S to soluble Li_2_S*_n_* is also a crucial part of the electrochemical redox system, as it promotes the utilization of active materials. The Li_2_S dissociation curve of *α*-Fe_2_O_3_ 400 mT shows a higher peak current, earlier peak emergence and greater Li_2_S dissociation capacity (Fig. 2b), indicating stronger Li_2_S-oxidation kinetics and higher sulfur utilization compared with those of *α*-Fe_2_O_3_ 0 mT (Fig. 2a). In addition, a significant Li_2_S residue is observed on the *α*-Fe_2_O_3_/CP (0 mT, inset of Fig. 2a), while it is more fully dissolved on the *α*-Fe_2_O_3_/CP (400 mT, inset of Fig. 2b). The EDS elemental mappings (Figs S5 and S6) of *α*-Fe_2_O_3_/CP (400 mT) show a smaller amount of sulfur element (0.28 wt%) than that of *α*-Fe_2_O_3_/CP (0 mT) (2.08 wt%) after Li_2_S dissociation (Table S1). These results suggest that the MF effectively promotes the conversion of Li_2_S to Li_2_S*_n_* (4 ≤ *n* ≤ 8). Furthermore, density functional theory (DFT) calculations were used to calculate the effect of the applied MF on the Fe d-band center of *α*-Fe_2_O_3_ (104)–Li_2_S, as shown in Fig. 2c and d. The projected density of states (PDOS) results demonstrate that the Fe d-band center value increases from −1.39 eV (without the MF) to −1.30 eV (with the MF) and the bandgap width decreases from 0.75 eV (without the MF) to 0.52 eV (with the MF). In addition, the hybridization degree (HD) between the Fe 3d and S 3p orbitals increases from 75.98% (without the MF) to 79.42% (with the MF). The results suggest that applying the MF can facilitate electron transfer and enhance the reaction kinetics of Li_2_S conversion.

**Figure 3.**
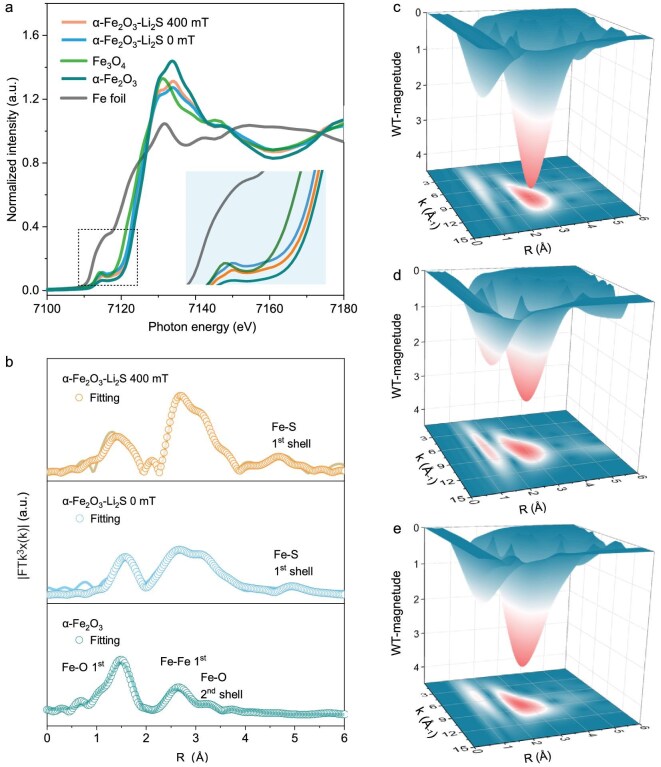
(a) Normalized Fe K-edge XANES spectra. (b) FT-EXAFS curves and fitting curves of *α*-Fe_2_O_3_–Li_2_S with 0/400-mT MF and pristine *α*-Fe_2_O_3_. (c–e) 3D WT-EXAFS contour maps of (c) *α*-Fe_2_O_3_–Li_2_S 400 mT, (d) *α*-Fe_2_O_3_–Li_2_S 0 mT and (e) pristine *α*-Fe_2_O_3_.

The local structure and coordination environment of *α*-Fe_2_O_3_–Li_2_S (after Li_2_S deposition with 400 and 0 mT) were examined by using synchrotron radiation-based X-ray absorption near-edge structure (XANES) spectra and Fourier-transformed extended X-ray absorption fine structure (FT-EXAFS). As shown in Fig. 3a, the XANES profiles of *α*-Fe_2_O_3_–Li_2_S 400 and 0 mT lie between those of Fe_3_O_4_ and *α*-Fe_2_O_3_, indicating that the average oxidation state of Fe is between those of the two reference samples. This can be attributed to the reduction of the iron valence induced by the sulfur-containing environment. As depicted in Fig. 3b, the FT-EXAFS fitting curves of *α*-Fe_2_O_3_–Li_2_S 400 mT and 0 mT show four characteristic peaks at about 1.5, 2.5, 3.2 and 5 Å, corresponding to the Fe–O first, Fe–Fe first, Fe–O second and Fe–S first coordinations, respectively. In this case, the sulfur atoms of Li_2_S do not replace oxygen atoms within the lattice of *α*-Fe_2_O_3_, and thus the Fe and S atoms in *α*-Fe_2_O_3_–Li_2_S form the long-range path Fe–S coordination, differing from that of the Fe–S chemical bond (2 Å) in the FeS_2_ powder (Figs S7 and S8). Instead, the sulfur atoms of Li_2_S are located farther away from the Fe atoms (5 Å). To obtain quantitative structural information, FT-EXAFS was fitted in the real-space (R-space), as shown in Fig. 3b and Table S2. Fe atoms in *α*-Fe_2_O_3_–Li_2_S 400 mT display a higher coordination number of 11.5 and a shorter Fe–S distance of 5.27 Å than those in *α*-Fe_2_O_3_–Li_2_S 0 mT (3.3 and 5.65 Å, respectively), due to the enhanced adsorption of Li_2_S on *α*-Fe_2_O_3_ under a 400-mT MF. The more powerful 3D and 2D wavelet transform EXAFS (WT-EXAFS) analysis of the Fe K-edge was conducted to provide high resolution in the k and R spaces, as shown in Fig. 3c–e and Figs S8–S10. As illustrated in Fig. 3c–e, all three samples exhibited maximum intensities at ∼5 and 8 Å^−1^ (k (Å^−1^) space), which are attributed to Fe–O and Fe–Fe, respectively. In contrast to the pristine *α*-Fe_2_O_3_ (Fig. 3e), the *α*-Fe_2_O_3_–Li_2_S 400 mT and 0 mT at 4.5–5.0 Å exhibited distinctly different coordination environments, with stronger WT oscillations and longer R-space coordination distances. This observed coordination region is attributed to Fe–S coordination (Fig. 3c and d), which is consistent with the R-space-fitting results (Fig. 3b). In summary, the coordination environment of *α*-Fe_2_O_3_ is significantly affected by the MF and Li_2_S deposition. Compared with a 0-mT MF, the application of a 400-mT MF results in a shorter Fe–S coordination distance, which facilitates the catalytic conversion of Li_2_S.

### MF-guided adsorption and catalysis toward LiPSs

**Figure 4.**
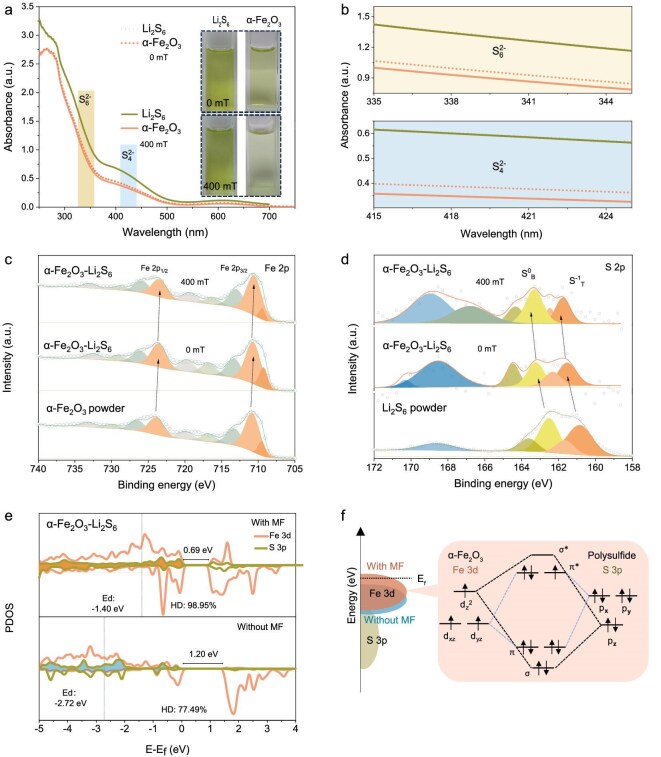
(a) Ultraviolet-visible (UV-vis) spectra for Li_2_S_6_ solution and that adsorbed by *α*-Fe_2_O_3_ after 2 h with MFs of 0 and 400 mT. The insets show the corresponding optical photos. (b) Magnified view of the absorbance curves in (a). (c) Fe 2p and (d) S 2p X-ray photoelectron spectroscopy spectra of *α*-Fe_2_O_3_ particles and *α*-Fe_2_O_3_–Li_2_S_6_ with MFs of 0 and 400 mT. (e) PDOS of Fe 3d and S 3p orbitals of *α*-Fe_2_O_3_–Li_2_S_6_ with and without MFs. (f) Schematic diagrams of the distribution center of the Fe d-band with and without MFs, and the orbitals interaction between the polysulfide and *α*-Fe_2_O_3_ with the MF.

Strong adsorption of polysulfides by the catalyst could mitigate the shuttle effect of long-chain polysulfides (Li_2_S*_n_*, 4 ≤ *n* ≤ 8) [42]. To study the effect of the MF on the adsorption of LiPSs by *α*-Fe_2_O_3_, the Li_2_S_6_-adsorption experiments were carried out with 0 and 400 mT of MF. After aging for 2 h, the Li_2_S_6_ solution containing *α*-Fe_2_O_3_ (*α*-Fe_2_O_3_–Li_2_S_6_) appeared more transparent, as shown in the insets of Fig. 4a. The two regions highlighted at 340 and 420 nm correspond to the absorption peaks of S_6_^2−^ and S_4_^2−^, respectively [43,44]. As shown in the magnified views in Fig. 4b, under a 400-mT MF, the peak intensities of S_6_^2−^ and S_4_^2−^ in the *α*-Fe_2_O_3_–Li_2_S_6_ solution are significantly reduced compared with those in the pure Li_2_S_6_ solution and the *α*-Fe_2_O_3_–Li_2_S_6_ 0 mT, indicating that the MF promotes the adsorption of LiPSs by *α*-Fe_2_O_3_.

To further investigate the MF-induced adsorption of the catalyst toward polysulfides, X-ray photoelectron spectroscopy (XPS) was employed to analyse the valence-state changes in Fe and S after the Li_2_S_6_-adsorption experiments, as shown in Fig. 4c and d. The initial peaks at 710.80 and 723.90 eV of the *α*-Fe_2_O_3_ particles are attributed to Fe 2p_3/2_ and Fe 2p_1/2_, respectively, which shift to lower binding energies of 710.69 and 723.59 eV after the adsorption of Li_2_S_6_ (*α*-Fe_2_O_3_–Li_2_S_6_, 0 mT), respectively. Moreover, the terminal sulfur (S_T_^−1^) and bridge sulfur (S_B_^0^) of the polysulfide in *α*-Fe_2_O_3_–Li_2_S_6_ 0 mT shift to higher binding energy compared with those of the Li_2_S_6_ powder. The decrease in the Fe valence and the increase in the S valence indicate the transfer of electrons from the LiPSs to *α*-Fe_2_O_3_ during the adsorption process. In contrast, under a 400-mT MF, the binding energy is increased by 0.24 and 0.08 eV for S_T_^−1^ and S_B_^0^, and decreased by 0.13 and 0.1 eV for Fe 2p_3/2_ and Fe 2p_1/2_, respectively, compared with that of *α*-Fe_2_O_3_–Li_2_S_6_ 0 mT, indicating that the MF enhances the chemical adsorption of Li_2_S_6_ by *α*-Fe_2_O_3_. XANES was utilized to characterize the S K-edge of *α*-Fe_2_O_3_–Li_2_S_6_ (with 0 and 400 mT of MF), as shown in Fig. S11. The results also show that the XANES energy of the S K-edge shifts towards higher energy after the MF is applied, implying electron transfer from Li_2_S_6_ to *α*-Fe_2_O_3_. These findings demonstrate that the MF enhances the chemical adsorption of Li_2_S_6_ by *α*-Fe_2_O_3_, consistent with the above XPS analysis.

DFT calculations were conducted to investigate the electron interactions between *α*-Fe_2_O_3_ and Li_2_S_6_. The optimized adsorption model of *α*-Fe_2_O_3_ (104)–Li_2_S_6_ is shown in Fig. S12. Figure 4e and Fig. S13 show the calculated density of states (DOS) and projected density of states (PDOS) for *α*-Fe_2_O_3_–Li_2_S_6_ with/without MF. In Fig. 4e, the bandgap width of *α*-Fe_2_O_3_–Li_2_S_6_ narrows from 1.20 to 0.69 eV after the MF is applied, indicating the improved conductivity of *α*-Fe_2_O_3_–Li_2_S_6_. In addition, the position of the metal d-band center relative to the Fermi energy level can be used to assess the adsorption and catalytic performances of the metal sites [45]. The Fe d-band center energy (*E*_d_) of *α*-Fe_2_O_3_–Li_2_S_6_ increases from −2.72 to −1.40 eV after the MF is applied. This shift involves an increase in the orbital energy, suggesting that electrons are more likely to adopt a high-spin configuration [18,46]. Based on the results of the PDOS calculations, a schematic diagram illustrating the orbital interactions between Fe^3+^ with a high-spin 3d electronic structure and the polysulfide under the MSF is shown in Fig. 4f. According to the molecular orbital theory and ligand field theory, the d*_xz_*, d*_yz_* orbitals of the Fe^3+^ in *α*-Fe_2_O_3_ form two π bonds and two π* anti-bonding orbitals with the p*_x_*, p*_y_* orbitals of the sulfur atoms in the polysulfide. The ${{\mathrm{d}}}_{{z}^2}$ orbital of Fe^3+^ with the p*_z_* orbital of S form σ bond and σ* anti-bonding orbitals. Additionally, the d*_xy_* and ${{\mathrm{d}}}_{{x}^2 - {y}^2}$ orbitals do not participate in bonding due to their inactivity compared with the other Fe^3+^ d states. Less electron filling in the anti-bonding orbitals (π* and σ*) facilitates the reduction of *α*-Fe_2_O_3_ and Li_2_S_6_ repulsion, enhancing the adsorption of polysulfides by *α*-Fe_2_O_3_ [22,23,47]. Specifically, the HD of the Fe 3d and S 3p orbitals were calculated over the range of −5 to 0 eV, as shown in Fig. 4e. The orbital HD can be used to measure electron interactions, where a high HD value indicates reduced electron repulsion with enhanced electron migration between the atoms and favors a catalytic effect [19,44,48]. The HD between the Fe 3d and S 3p orbitals is defined as $\frac{{DO{S}_{{\mathrm{overlapped}}}}}{{DO{S}_{{\mathrm{S\ }}3{\mathrm{p\ states}}}}}$, where *DOS*_overlapped_ and *DOS*_S 3p states_ are the integration of the overlapping Fe 3d with the S 3p states and the total S 3p states, respectively [49]. The HD of *α*-Fe_2_O_3_–Li_2_S_6_ is 98.95% with the MF and 77.49% without the MF, respectively. This indicates that the MF promotes the electron transfer between the Fe atoms of *α*-Fe_2_O_3_ and the S atoms of Li_2_S_6_, which can effectively enhance the conversion activity of the LiPS, reduce the LiPS shuttle effect and improve the utilization efficiency of the active material.

### Thermodynamically understanding Li_2_S deposition/dissociation and the polysulfide conversion

**Figure 5.**
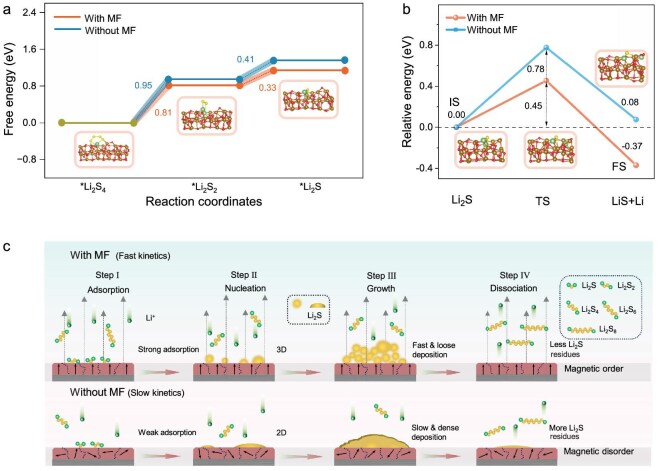
(a) Gibbs free energy for the discharging process from Li_2_S_4_ to Li_2_S on *α*-Fe_2_O_3_ with and without an MF. Optimized absorption configurations with the MF are shown in the insets. (b) Energy-barrier profiles of Li_2_S decomposition on *α*-Fe_2_O_3_ catalysts along with different reaction coordinates with and without MFs. Initial, transition and final states (IS, TS, FS) with MF are shown in the insets. (c) Schematic illustration of the catalytic mechanism of *α*-Fe_2_O_3_ catalysts involving the adsorption, nucleation, growth and dissociation of Li_2_S with and without MFs.

It is well known that, in Li–S batteries, the conversion reactions from Li_2_S_4_ to Li_2_S (Li_2_S_4_→Li_2_S_2_→Li_2_S) involve a liquid–solid transition (Li_2_S_4_→Li_2_S_2_) and a solid–solid transition (Li_2_S_2_→Li_2_S). The latter has a high energy barrier and serves as the rate-determining step that affects the discharge capacity of Li–S batteries. DFT Gibbs free energy calculations, as shown in Fig. 5a and Fig. S14, demonstrate the facilitating effect of the MF on the conversion of short-chain polysulfides from Li_2_S_4_ to Li_2_S, in which both the Li_2_S_4_→Li_2_S_2_ and the Li_2_S_2_→Li_2_S transformations under the MF have lower Gibbs free energies compared with those without the MF, leading to a faster reaction rate with the MF. In addition, the delithiation kinetics of Li_2_S during the charging process is also a factor that determines the reversibility of Li–S batteries. Thus, modeling of the decomposition of Li_2_S was performed to assess the reaction kinetics. The intrinsic kinetics barrier in the reaction can be deduced from the dissociation energy of Li_2_S into LiS and Li atoms. The optimized structures of the decomposition of Li_2_S on *α*-Fe_2_O_3_ catalysts with/without an MF and their energy barriers are shown in Fig. 5b and Fig. S15. It is obvious that the Li_2_S delithiation reaction under the MF has a lower energy barrier (0.45 eV) compared with that without the MF (0.78 eV). The decreased energy barrier is consistent with the experimentally observed more complete dissociation of Li_2_S on the *α*-Fe_2_O_3_/CP (400-mT MF) (Fig. 2b). Based on the above experimental and calculated results, we propose a mechanism for the conversion of short-chain polysulfides into Li_2_S and the dissociation of Li_2_S into long-chain polysulfides on *α*-Fe_2_O_3_ catalysts with/without an MF, as shown in Fig. 5c.

With an MF, *α*-Fe_2_O_3_ undergoes the consistent alignment of magnetic-domain orientations, which facilitates electron migration between the *α*-Fe_2_O_3_ catalyst and the LiPSs (Fig. 5c, Step I). Besides, the Fe d-band center moves closer to the Fermi level, along with the enhanced hybridization between the Fe 3d and S 3p orbitals, which strengthens the adsorption of polysulfides on *α*-Fe_2_O_3_ and accelerates catalytic reaction kinetics to form Li_2_S molecules that are deposited on the surface of the *α*-Fe_2_O_3_ on the electrodes (Step I). Subsequently, the deposited Li_2_S, as nucleation sites, capture LiPSs near the electrolyte–Li_2_S interface, followed by disproportionation to continuously generate Li_2_S (Step II) [49]. Due to the faster catalytic reaction kinetics of the MF-guided catalyst toward both LiPSs and solid Li_2_S (as shown in Fig. 2d), the 3D nucleation and growth mode were favored over the slower lateral diffusion mode (Fig. 2b) (Step II). Consequently, the rapid nucleation and growth of Li_2_S triggers the dynamically controlled deposition process, forming a loosely stacked Li_2_S layer (Step III). During the charging process, the dissociation of Li_2_S under the MF exhibits a lower energy barrier, facilitating its conversion into long-chain polysulfides, with a lower amount of Li_2_S remaining (Step IV). In contrast, without the applied MF, the magnetically disordered *α*-Fe_2_O_3_ catalyst shows weak adsorption and sluggish catalytic reaction kinetics towards the LiPSs and Li_2_S, rendering a 2D nucleation and growth mode (Fig. 5c, Step I and Step II). As a result, Li_2_S suffers from a slow and dense deposition during the conversion reaction, which is a thermodynamically dominated process, leading to the formation of an energy-stable passivation film (Step III). Accordingly, more Li_2_S residues are generated after the dissociation of the passivation film during the charging process without an MF (Step IV). The above results are confirmed by the SEM and EDS characterizations shown in Fig. 2a, b, Figs S5 and S6. Overall, the MF enhances the adsorption and conversion kinetics of polysulfides on *α*-Fe_2_O_3_, promotes the 3D nucleation and growth of Li_2_S, and reduces the energy barrier for Li_2_S dissociation.

### Electrochemical performance of *α*-Fe_2_O_3_/S cathode and system expansion

**Figure 6.**
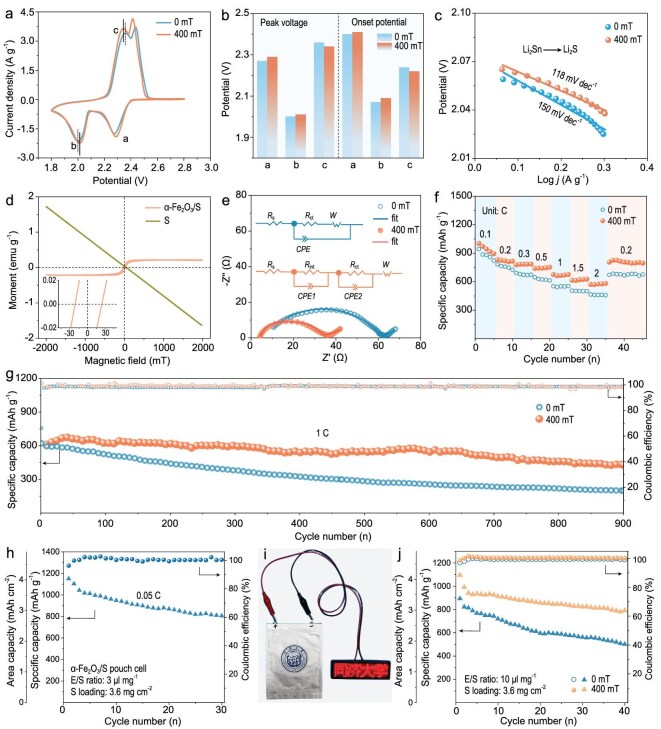
(a) CV curves and (b) peak voltages and onset potentials of Points a, b and c on CV curves. (c) Tafel plots of Peak b of *α*-Fe_2_O_3_/S with MFs of 0 and 400 mT. (d) Magnetic hysteresis loops of *α*-Fe_2_O_3_/S and sulfur. (e) EIS plots. *R*_s_, internal resistance including the resistances of the electrolyte solution and electrodes; *R*_ct_, charge-transfer resistance; *R*_int_, interphase resistance; *W*, Warburg resistance; *CPE*, capacitance contributed by the charge-transfer process. (f ) Rate performances and (g) long-cycle tests at 1 C of *α*-Fe_2_O_3_/S with MFs of 0 and 400 mT. (h) Cycling stability of *α*-Fe_2_O_3_/S pouch cell at 0.05 C under MF of 0 mT. (i) Digital photo of a light emitting diode (LED). (j) Cycling stability of *α*-Fe_2_O_3_/S cathodes with high sulfur loading of 3.6 mg cm^−2^ at 0.05 C.

*α*-Fe_2_O_3_/S cathodes were assembled to investigate the effect of the MF on the electrochemical performance of Li–S batteries. The SEM image, EDS element mappings and XRD patterns of the *α*-Fe_2_O_3_/S cathode are shown in Figs S16 and S17, confirming that *α*-Fe_2_O_3_ and S are uniformly mixed in *α*-Fe_2_O_3_/S. Figure 6a presents the cyclic voltammetry (CV) curves of *α*-Fe_2_O_3_/S at 0.2 mV s^−1^ under 0 and 400 mT of MF. In this work, the MF was generated by using a permanent magnet (Fig. S18). Two reduction peaks appear at ∼2.3 V (Peak a) and ∼2.0 V (Peak b), which can be attributed to the formation of long-chain LiPSs and insoluble short-chain Li_2_S*_n_* (*n* = 1, 2), respectively. Meanwhile, the oxidation peaks at ∼2.4 V correspond to the oxidation of Li_2_S*_n_*. Peaks a, b and c of the CV curves were further analysed, as shown in Fig. 6b. Peaks a and b of the CV curves show higher peak voltages and onset voltages under the 400-mT MF, which suggests that the *α*-Fe_2_O_3_/S with a 400-mT MF has a higher discharge plateau and smaller voltage polarization during the discharge. Peak c at 400 mT has lower peak voltage and onset voltage than those of 0 mT, indicating lower charging voltages and smaller voltage polarization, which suggests that Li_2_S dissociation has faster conversion kinetics under the 400-mT MF. The voltage polarization of Peaks a, b and c is clearly presented by the Tafel plots (Fig. 6c, Fig. S19a and b), which show smaller Tafel slopes under the 400-mT MF. The results suggest that the MF-guided Fe_2_O_3_ catalytic system is conducive to promoting the conversion kinetics of polysulfides and Li_2_S. The magnetic hysteresis loops of the *α*-Fe_2_O_3_/S and sulfur powders were characterized by using a VSM, as shown in Fig. 6d. A significant decrease in the saturation magnetization of *α*-Fe_2_O_3_/S (0.21 emu g^−1^) is observed compared with that of *α*-Fe_2_O_3_ (1.11 emu g^−1^, Fig. 1c), which is due to the diamagnetic nature of sulfur. The coercivity slightly increases from 16.9 mT (*α*-Fe_2_O_3_) to 21.3 mT (*α*-Fe_2_O_3_/S), as shown in the inset of Fig. 6d. The first-cycle charge/discharge curves at 0.1 C (Fig. S20) also exhibit a smaller voltage polarization of 0.18 V under 400 mT compared with 0.21 V under 0 mT. Note that the *α*-Fe_2_O_3_/S 400 mT shows a larger current response, higher specific capacity and lower voltage polarization as compared with *α*-Fe_2_O_3_/S 0 mT, which suggests that the MF enhances the redox kinetics throughout the *α*-Fe_2_O_3_-catalysed conversion of polysulfides and Li_2_S. As shown in Fig. 6e, the equivalent circuit of electrochemical impedance spectroscopy (EIS) was used to simulate each circuit element. The charge-transfer resistance (*R*_ct_) of *α*-Fe_2_O_3_/S 400 mT (28.03 Ω) is lower than that of *α*-Fe_2_O_3_/S 400 mT (53.96 Ω), while the internal impedance (*R*_s_) is decreased from 6.52 Ω (0 mT) to 4.11 Ω (400 mT), indicating that the MF can facilitate electron migration. The EIS results are consistent with the previous DFT calculations (Figs 4e and 2d), confirming that the MF reduces the bandgap widths of *α*-Fe_2_O_3_–Li_2_S_6_ and *α*-Fe_2_O_3_–Li_2_S, thereby promoting electron transfer between *α*-Fe_2_O_3_ and Li_2_S_6_/Li_2_S.

Meanwhile, Fig. 6f illustrates the rate performances of the *α*-Fe_2_O_3_/S cathode under 0/400 mT of MF, in which it can be observed that all the specific capacities of *α*-Fe_2_O_3_/S 400 mT exceed those of 0 mT from 0.1 to 2 C. Upon a return to 0.2 C, the *α*-Fe_2_O_3_/S 400 mT demonstrates an average reversible capacity of 807 mAh g^−1^ (388 mAh g^−1^, based on the mass of the entire cathode). *α*-Fe_2_O_3_/S 400 mT also shows a higher average capacity and more stable long cycling performance, as shown in Fig. 6g. In the initial cycles, *α*-Fe_2_O_3_/S 400 mT undergoes an activation state, resulting in a slight increase in the specific capacity. The capacity decay rate per cycle for *α*-Fe_2_O_3_/S 400 mT is only 0.018%, which is significantly lower than 0.076% observed for *α*-Fe_2_O_3_/S 0 mT. After 900 cycles at 1 C, *α*-Fe_2_O_3_/S 400 mT maintains a higher capacity of 431 mAh g^–1^ compared with that of 0 mT. High sulfur loading is a key parameter for achieving high energy density in Li–S batteries. In Fig. S21, under 400 mT, the battery with an *α*-Fe_2_O_3_/S cathode and a sulfur loading of 4.8 mg cm^−2^ (E/S = 12 μL mg^−1^) exhibits an average capacity of 690 mAh g^−1^ over 50 cycles at 0.05 C, which is significantly higher than 494 mAh g^−1^ under 0 mT. This electrochemical performance demonstrates that the MF favors the capacity enhancement and cycling stabilization of *α*-Fe_2_O_3_/S.

Cathodes with high sulfur content (60 wt%) and high areal sulfur loading (3.6 mg cm^−2^) were assembled into both coin cells and pouch cells. These cells demonstrated high specific capacity and excellent cycling stability even in the absence of an MF (Fig. 6h and i, Fig. S22 and Table S3).

As depicted in Fig. 6h, the initial specific capacity of the *α*-Fe_2_O_3_/S cathode in the pouch cell was 1150 mAh g^−1^ (693 mAh g^−1^, based on the mass of the entire cathode) at 0.05 C without an MF. This indicates that the pouch cell can deliver an energy density of 283 Wh kg^−1^. Subsequently, the coin cell was tested under a 400-mT MF. It delivered an initial specific capacity of 1095 mAh g^−1^ (660 mAh g^−1^, based on the mass of the entire cathode) and retained 791 mAh g^−1^ after 40 cycles (Fig. 6j). This performance is superior to that of the high-loading coin cell tested at 0 mT, showing enhanced specific capacity and cycling stability under the MF. Furthermore, KB/S (Ketjen black/sulfur, without an *α*-Fe_2_O_3_ catalyst) coin cells were assembled for rate performance testing. A minor performance improvement was observed under the MF; however, the enhancement was significantly more pronounced for the catalyst-containing cathode (*α*-Fe_2_O_3_/S), as clearly evidenced by the data shown in Figs S23, S24 and Table S4. Collectively, these results substantiate the practical application potential of the *α*-Fe_2_O_3_/S cathode under an external MF.

The MF-guided catalysis strategy has also been applied to a ferromagnetic nano-Fe_3_O_4_ catalyst-based system. The SEM image and magnetic hysteresis loop of Fe_3_O_4_ powder are shown in Fig. S25a and b. The adsorption of Li_2_S_6_ by the Fe_3_O_4_ powder was carried out as shown in Fig. S26a and b. These data indicate that MF promotes the adsorption of long-chain polysulfides by Fe_3_O_4_. The Li_2_S-deposition capacity shows a significant increase from 438 mAh g^−1^ (0 mT) to 519 mAh g^−1^ (400 mT), as shown in Fig. S27a and b. Additionally, as shown in the insets of Fig. S27a and b, Li_2_S exhibits a reticular structure at point A' of the deposition curve, while a 2D passivation film (dashed yellow curve, points A and B) is observed. At the endpoints (B and B') of the Li_2_S-deposition curves, an irregular multi-particle appearance is observed. However, Fe_3_O_4_/CP 400 mT shows looser and larger Li_2_S particles. In addition, Fe_3_O_4_/S was used as the cathode for the Li–S batteries. The CV curves, charge/discharge curves and rate performance of Fe_3_O_4_/S show that the MF reduced the voltage polarization, promoted the kinetics of the polysulfides and Li_2_S conversion, and enhanced the capacity and cycling stability (Figs S28a, b and S29).

## CONCLUSION

In this work, we investigate the effect of an MF on the catalytic ability of magnetic Fe-based oxides in Li–S batteries. MF-guided catalysis by *α*-Fe_2_O_3_ promotes not only polysulfides conversion, but also Li_2_S deposition/dissociation, leading to the formation of a highly electroactive loose film, synchronously realizing shuttle-effect suppression and Li_2_S anti-passivation for high-performance Li–S batteries. The mechanism stems from the fact that the MF can enhance the electron spin polarization of the Fe 3d orbital in *α*-Fe_2_O_3_, shifting the center of the Fe d-band towards the Fermi level and increasing the hybridization between the Fe 3d and S 3p orbitals in *α*-Fe_2_O_3_–Li_2_S_6_ or *α*-Fe_2_O_3_–Li_2_S. As a result, under a 400-mT MF, the *α*-Fe_2_O_3_/S cathode shows outstanding cycling stability (≤900 cycles at 1 C with a 0.018% capacity decay rate per cycle) and excellent rate capability.

## Supplementary Material

nwag039_Supplemental_File

## References

[1] Yin Y, Xin S, Guo Y et al. Lithium–sulfur batteries: electrochemistry, materials, and prospects. Angew Chem Int Ed 2013; 52: 13186–200. 10.1002/anie.201304762 24243546

[2] Zhang C, Lu X, Han X et al. Identifying the role of the cationic geometric configuration in spinel catalysts for polysulfide conversion in sodium–sulfur batteries. J Am Chem Soc 2023; 145: 18992–9004. 10.1021/jacs.3c06288 37603793

[3] Zhang X, Zhang X, Wang X et al. Engineering spin states of metal sites toward advanced lithium–sulfur batteries. Energy Environ Sci 2025; 18: 3553–67. 10.1039/D4EE05582A

[4] Song Y, Zhou J, Chen Z et al. Reducing the cathode Thiele modulus to promote the discharge capacity of lithium–sulfur batteries. J Energy Chem 2025; 106: 993–1001. 10.1016/j.jechem.2025.04.075

[5] Li X, Feng S, Zhao M et al. Surface gelation on disulfide electrocatalysts in lithium-sulfur batteries. Angew Chem Int Ed 2022; 61: e202114671. 10.1002/anie.202114671 34889012

[6] Jin T, Li X, Zhao M et al. Promoting the rate performances of weakly solvating electrolyte-based lithium‒sulfur batteries. Angew Chem Int Ed 2025; 64: e202504898. 10.1002/anie.202504898 40458931

[7] Yu XF, Shao DY, Xu J et al. Recent advances in carbon-based sulfur host materials for lithium-sulfur batteries. Microstructures 2024; 4: 2024030. 10.20517/microstructures.2023.82

[8] Gao R, Zhang M, Han Z et al. Unraveling the coupling effect between cathode and anode toward practical lithium–sulfur batteries. Adv Mater 2024; 36: 2303610. 10.1002/adma.202303610 37500064

[9] Wang P, Dai X, Xu P et al. Hierarchical and lamellar porous carbon as interconnected sulfur host and polysulfide-proof interlayer for Li–S batteries. eScience 2023; 3: 100088. 10.1016/j.esci.2022.100088

[10] Zhou J, Liu X, Zhu L et al. Deciphering the modulation essence of p bands in Co-based compounds on Li-S chemistry. Joule 2018; 2: 2681–93. 10.1016/j.joule.2018.08.010

[11] Chen Z, Zhao J, Fang G et al. Sufficient cathode infiltration for stable 500 Wh kg^−1^ level lithium–sulfur batteries. J Energy Chem 2025; 109: 129–37. 10.1016/j.jechem.2025.05.006

[12] Meng R, Du Q, Zhong N et al. A tandem electrocatalysis of sulfur reduction by bimetal 2D MOFs. Adv Energy Mater 2021; 11: 2102819. 10.1002/aenm.202102819

[13] Zhu Z, Zeng Y, Pei Z et al. Bimetal-organic framework nanoboxes enable accelerated redox kinetics and polysulfide trapping for lithium-sulfur batteries. Angew Chem Int Ed 2023; 62: e202305828. 10.1002/anie.202305828 37278545

[14] Liu Y, Meng X, Wang Z et al. Development of quasi-solid-state anode-free high-energy lithium sulfide-based batteries. Nat Commun 2022; 13: 4415. 10.1038/s41467-022-32031-7 35906196 PMC9338099

[15] Zhang M, Zhang K, Wei W et al. Arginine modification of hybrid cobalt/nitrogen Ti_3_C_2_T_x_ MXene and its application as a sulfur host for lithium-sulfur batteries. Microstructures 2024; 4: 2024013. 10.20517/microstructures.2023.68

[16] Jiang J, Ontaneda J, Pal S et al. Enhanced polysulfide trapping in Li–S batteries by dipole alignment in ferroelectric BaTiO_3_. Energy Environ Sci 2024; 17: 6291–301. 10.1039/D4EE01936A

[17] Yan J, Wang Y, Zhang Y et al. Direct Magnetic reinforcement of electrocatalytic ORR/OER with electromagnetic induction of magnetic catalysts. Adv Mater 2021; 33: 2007525. 10.1002/adma.202007525 33336466

[18] Zhao Y, Geng C, Wang L et al. Engineering catalytic defects via molecular imprinting for high energy Li-S pouch cells. Natl Sci Rev 2024; 11: nwae190. 10.1093/nsr/nwae190 38938275 PMC11210504

[19] Huang C, Yu J, Zhang CY et al. Electronic spin alignment within homologous NiS_2_/NiSe_2_ heterostructures to promote sulfur redox kinetics in lithium-sulfur batteries. Adv Mater 2024; 36: 2400810. 10.1002/adma.202400810 38569213

[20] Yan R, Zhao Z, Cheng M et al. Origin and acceleration of insoluble Li_2_S_2_-Li_2_S reduction catalysis in ferromagnetic atoms-based lithium-sulfur battery cathodes. Angew Chem Int Ed 2023; 62: e202215414. 10.1002/anie.202215414 PMC10107143 36321878

[21] Kang D, Zhang C, Wang X et al. Efficient atomically dispersed Fe catalysts with robust three-phase interface for stable seawater-based zinc-air batteries. Green Carbon 2025; 3: 1–10. 10.1016/j.greenca.2024.09.002

[22] Wang Z, Huang W, Wu H et al. 3d-orbital high-spin configuration driven from electronic modulation of Fe_3_O_4_/FeP heterostructures empowering efficient electrocatalyst for lithium-sulfur batteries. Adv Funct Mater 2024; 34: 2409303. 10.1002/adfm.202409303

[23] Zhang CY, Gong L, Zhang C et al. Sodium-sulfur batteries with unprecedented capacity, cycling stability and operation temperature range enabled by a CoFe_2_O_4_ catalytic additive under an external magnetic field. Adv Funct Mater 2023; 33: 2305908. 10.1002/adfm.202305908

[24] Liu R, Wei Z, Peng L et al. Establishing reaction networks in the 16-electron sulfur reduction reaction. Nature 2024; 626: 98–104. 10.1038/s41586-023-06918-4 38297176

[25] Zhang CY, Zhang C, Pan JL et al. Surface strain-enhanced MoS_2_ as a high-performance cathode catalyst for lithium–sulfur batteries. eScience 2022; 2: 405–15. 10.1016/j.esci.2022.07.001

[26] Ge J, Ren X, Chen RR et al. Multi-domain versus single-domain: a magnetic field is not a must for promoting spin-polarized water oxidation. Angew Chem Int Ed 2023; 62: e202301721. 10.1002/anie.202301721 37130000

[27] Wang Y, Meng P, Yang Z et al. Regulation of atomic Fe-Spin state by crystal field and magnetic field for enhanced oxygen electrocatalysis in rechargeable zinc-air batteries. Angew Chem Int Ed 2023; 62: e202304229. 10.1002/anie.202304229 37139572

[28] Shen K, Wang Z, Bi X et al. Magnetic field-suppressed lithium dendrite growth for stable lithium-metal batteries. Adv Energy Mater 2019; 9: 1900260. 10.1002/aenm.201900260

[29] Wang A, Deng Q, Deng L et al. Eliminating tip dendrite growth by Lorentz force for stable lithium metal anodes. Adv Funct Mater 2019; 29: 1902630. 10.1002/adfm.201902630

[30] Zhang W, Gao J, Huang Y et al. Utilizing magnetic-field modulation to efficiently improve the performance of LiCoO_2_||graphite pouch full batteries. Adv Funct Mater 2023; 33: 2306354. 10.1002/adfm.202306354

[31] Wang XX, Guan DH, Li F et al. Magnetic and optical field multi-assisted Li–O_2_ batteries with ultrahigh energy efficiency and cycle stability. Adv Mater 2022; 34: 2104792. 10.1002/adma.202104792 35023599

[32] Huang Y, Li Z, Zhu T et al. Ferromagnetic 1D-Fe_3_O_4_@C microrods boost polysulfide anchoring for lithium–sulfur batteries. ACS Appl Energy Mater 2021; 4: 3921–7. 10.1021/acsaem.1c00298

[33] Gao Z, Schwab Y, Zhang Y et al. Ferromagnetic nanoparticle–assisted polysulfide trapping for enhanced lithium–sulfur batteries. Adv Funct Mater 2018; 28: 1800563. 10.1002/adfm.201800563

[34] Li W, Liang Z, Lu Z et al. Magnetic field-controlled lithium polysulfide semiliquid battery with ferrofluidic properties. Nano Lett 2015; 15: 7394–9. 10.1021/acs.nanolett.5b02818 26422674

[35] Zhang CY, Zhang CQ, Sun G et al. Spin effect to promote reaction kinetics and overall performance of lithium-sulfur batteries under external magnetic field. Angew Chem Int Ed 2022; 61: e202211570. 10.1002/anie.202211570 36216781

[36] Zheng CY, Zhang C, Sun GW et al. Propelling polysulfides transformation for high-rate and long-life lithium–sulfur batteries. Nano Energy 2017; 33: 306–12. 10.1016/j.nanoen.2017.01.040

[37] Teja AS, Koh PY. Synthesis, properties, and applications of magnetic iron oxide nanoparticles. Prog Cryst Growth Charact Mater 2009; 55: 22–45. 10.1016/j.pcrysgrow.2008.08.003

[38] Chen RR, Chen G, Ren X et al. SmCo_5_ with a reconstructed oxyhydroxide surface for spin-selective water oxidation at elevated temperature. Angew Chem Int Ed 2021; 60: 25884–90. 10.1002/anie.202109065 34561927

[39] Sun J, Liu Y, Liu L et al. Interface engineering toward expedited Li_2_S deposition in lithium–sulfur batteries: a critical review. Adv Mater 2023; 35: 2211168. 10.1002/adma.202211168 36756778

[40] Wang B, Wang L, Ding D et al. Zinc-assisted cobalt ditelluride polyhedra inducing lattice strain to endow efficient adsorption-catalysis for high-energy lithium–sulfur batteries. Adv Mater 2022; 34: 2204403. 10.1002/adma.202204403 36208086

[41] Fan F, Carter W, Chiang Y. Mechanism and kinetics of Li_2_S precipitation in lithium–sulfur batteries. Adv Mater 2015; 27: 5203–9. 10.1002/adma.201501559 26257297

[42] Shen Z, Jin X, Tian J et al. Cation-doped ZnS catalysts for polysulfide conversion in lithium–sulfur batteries. Nat Catal 2022; 5: 555–63. 10.1038/s41929-022-00804-4

[43] Tong Z, Huang L, Liu H et al. Defective graphitic carbon nitride modified separators with efficient polysulfide traps and catalytic sites for fast and reliable sulfur electrochemistry. Adv Funct Mater 2021; 31: 2010455. 10.1002/adfm.202010455

[44] Li W, Qian J, Zhao T et al. Boosting high-rate Li–S batteries by an MOF-derived catalytic electrode with a layer-by-layer structure. Adv Sci 2019; 6: 1802362. 10.1002/advs.201802362 PMC6702624 31453053

[45] Liu G, Zeng Q, Sui X et al. Modulating d-band electronic structures of molybdenum disulfide via p/n doping to boost polysulfide conversion in lithium-sulfur Batteries. Small 2023; 19: 2301085. 10.1002/smll.202301085 37194979

[46] Li H, Chuai M, Xiao X et al. Regulating the spin state configuration in bimetallic phosphorus trisulfides for promoting sulfur redox kinetics. J Am Chem Soc 2023; 145: 22516–26. 10.1021/jacs.3c07213 37788438

[47] Shen J, Xu X, Liu J et al. Unraveling the catalytic activity of Fe–based compounds toward Li_2_S_x_ in Li–S chemical system from d–p bands. Adv Energy Mater 2021; 11: 2100673. 10.1002/aenm.202100673

[48] Yan M, Dong W, Liu F et al. Unprecedented strong and reversible atomic orbital hybridization enables a highly stable Li–S battery. Natl Sci Rev 2022; 9: nwac078. 10.1093/nsr/nwac078 35832774 PMC9273299

[49] Li J, Zheng C, Zhao E et al. Ferromagnetic ordering correlated strong metal–oxygen hybridization for superior oxygen reduction reaction activity. Proc Natl Acad Sci USA 2023; 120: e2307901120. 10.1073/pnas.2307901120 37844253 PMC10614601

