# Supplementary material for: Magnetic-field-guided catalytic effect mitigates Li2S passivation of lithium–sulfur batteries
Source: Natl Sci Rev. 2026 Jan 19;13(6):nwag039. doi: 10.1093/nsr/nwag039 (PMC13017786; doi:10.1093/nsr/nwag039)
Supplement: nwag039_Supplemental_File [file nwag039_supplemental_file.pdf]

Supplemental Information

Magnetic field-guided catalytic effect mitigates Li_2S passivation of lithium-sulfur batteries

Lang Liao[1,3], Ruijin Meng[1]*, Chen Zhou[1], Shusheng Li[1], Wei Xu[4], Xiaoning Li[5], Kai Zhang[6], Lu Chen[1], Fangyi Shi[7], Duanzijing Liu[7], Hongying Hou[3,]*, Chi Zhang[1]* and Jinhu Yang[1,2]*

[1] School of Chemical Science and Engineering, and State Key Laboratory of Cardiovascular Diseases, Shanghai East Hospital, Tongji University, Shanghai 200092, China

[2] College of Materials and Chemical Engineering, Fuyang University of Technology, Anhui 236115, P. R. China

[3] Faculty of Material Science and Engineering, Kunming University of Science and Technology, Southwest United Graduate School, Kunming 650092, China

Kunming 650093, China

[4] Shanghai Eye Diseases Prevention & Treatment Center/Shanghai Eye Hospital, School of Medicine, Tongji University; National Clinical Research Center for Eye Diseases; Shanghai Engineering Research Center of Precise Diagnosis and Treatment of Eye Diseases, Shanghai, China

[5] Centre for Atomaterials and Nanomanufacturing (CAN), School of Science, RMIT University, Melbourne, VIC 3000, Australia

[6] Frontiers Science Center for New Organic Matter, State Key Laboratory of Advanced Chemical Power Sources, Key Laboratory of Advanced Energy Materials Chemistry (Ministry of Education), Collaborative Innovation Center of Chemical Science and Engineering (Tianjin), College of Chemistry, Nankai University, Tianjin 300071, P. R. China.

[7] Department of Applied Physics, the Hong Kong Polytechnic University, Hunghom, Hong Kong

* Corresponding author. E-mail: ruijinmeng@hnu.edu.cn; hongyinghou@kust.edu.cn; chizhang@tongji.edu.cn; yangjinhu@tongji.edu.cn

Experimental Section

Chemicals

α-Fe_2O_3 (99.5%, MACKLIN), 1, 2-dimethoxyethane (DME, 99.9%, DoDoChem), sulfur (S_8, 99.5%, Aladdin) and lithium sulfide (Li_2S, 99.9%, MACKLIN). All chemical reagents were used as received without further processing.

Li_2S_6 absorption experiments

The 0.2 mmol L^{-1} (mM) Li_2S_6 solution was prepared by adding 18.38 mg Li_2S, 64.12 mg S_8 and 10 mL DME to a sample bottle, then the bottle was heated at 65 ℃ with stirring for 48 h in an Ar filled glove box. Additionally, 5 mg α-Fe_2O_3 was added to 3 mL 0.2 mM Li_2S_6 solution, after resting for 2 h, the supernatant was taken for UV-visible adsorption characterization. Using the above same method, a parallel test was performed except that 400 mT magnet was placed under the sample bottle to demonstrate the effect of the MF on the Li_2S_6 adsorption.

Li_2S nucleation and dissociation tests

The 0.2 mol L^{-1} Li_2S_8 catholyte was prepared by mixing Li_2S and S_8 in DME solvent with a molar ratio of 1: 7. Commercial carbon paper (CP) (diameter 10 mm) as cathode collector and a Li foil (diameter 16 mm, thickness 0.5 mm) served as anode. Li_2S_8 catholyte (20 μL, 0.2 mol L^{-1}) was dropped into the cathode and LiTFSI (20 μL, 1.0 mol L^{-1}) anolyte was added to the Li anode. The Li_2S nucleation test was mainly divided into two processes: galvanostatically discharged to 2.12 V at 0.112 mA, second, it was potentiostatically discharged at 2.05 V until the current was below 10^{-5} A.

For the Li_2S dissociation test, it was first galvanostatically discharged to 1.7 V at 0.1 mA, then discharged at 0.01 mA until the voltage was below 1.8 V. Next, potentiostatically charged at 2.4 V until the current was below 10^{-5} A. The batteries were disassembled in an argon-filled glove box. The cathode electrodes were removed and cleaned with DME. Scanning electron microscope (SEM) images of Li_2S deposition/dissociation on CP were captured after the electrode dried.

Assemble of α-Fe_2O_3 pouch cell

A 100 μm lithium foil was used as the anode; a microporous PP membrane was used

as the separator; an α-Fe_2O_3/S electrode (4 cm × 5 cm) with a S loading of 3.6 mg cm^{-2} as the cathode, with an S content 60 wt%. The E/S ratio was set at 3 μL mg^{-1}. Before the electrolyte was injected, the pouch cell was cold-pressed tightly at 0.05 MPa. The cell was kept at 25 ℃ for 2 h under vacuum and then packaged in a vacuum chamber. After resting for 12 h, the assembled Li-S pouch cell was cycled at 0.05 C using a LAND battery tester. The energy density of pouch cell was calculated based on the total mass of constituent materials within the cell, including the cathode, anode, electrolyte, and separator.

Characterizations

The morphology of these samples was observed using a SEM (TESCAN Mira3) equipped with energy-dispersive X-ray spectroscopy (EDS). X-ray diffraction (XRD) patterns were obtained by using a D8 advance X-ray diffractometer with a Cu Kα radiation source (λ = 0.15418 nm). The magnetization curve was obtained using an Apparatus LakeShore 8604 with a magnetic field range ±1.5 T at room temperature. X-ray photoelectron spectroscopy (XPS, AXIS SUPRA) was used to analyze the composition and valence states of materials. X-ray absorption near edge structure spectra (S K-edge) were collected at 4B7A station in Beijing Synchrotron Radiation Facility (BSRF). The storage rings of BSRF were operated at 2.5 GeV with a maximum current of 250 mA. Using Si (111) double-crystal monochromator. The X-ray absorption near-edge structure (XANES) and extended X-ray absorption fine structure (EXAFS) spectra of the Fe K-edge were collected at the X-ray absorption spectroscopy beamline at the Australian Synchrotron (AS, Australia).

Electrochemical measurements

The sulfur composites were obtained by mixing catalyst, conductive agent (CMK-3), and sulfur in a ratio of 3: 1: 6 and heating at 155 ℃ for 12 h inside a muffle furnace. The cathode sheet was obtained by mixing and grinding the sulfur composite material, conductive agent (Super P), and binder (PVDF) in a ratio of 8: 1: 1, coating the hydrophilic carbon paper (CP) with a thickness of 0.2 mm by a spatula, and drying the cathode in a vacuum oven at 60 ℃ for 12 h to obtain a normal loading sulfur mass of 1~1.2 mg cm^{-2}. The obtained electrode was assembled with a Celgard separator, 0.5

mm Li and commercial Li-S electrolyte (1.0 M LiTFSI dissolved in a mixed solvent of DME and DOL (1: 1 in volume, 2 wt % $LiNO_3$)). The above electrolyte was dropped on both sides of the separator according to the ratio of electrolyte to sulfur E/S= 30 μL mg^{-1} (normal loading) and 12 μL mg^{-1} (high loading) for CR2025 coin batteries in an Ar filled glove box. The batteries were charged and discharged using a Neware charge/discharge instrument, and cyclic voltammetry (CV) tests (1.8 V ~ 2.8 V) and electrochemical impedance (EIS) tests (frequency range: 1 MHz ~ 0.1 Hz, amplitude 0.005 V) tests were performed using a CHI660 electrochemical workstation.

Scharifker-Hills Li_2S theoretical nucleation models

The patterns of Li_2S deposition can be determined by comparing the nondimensionalized current–time profile using a series of ready-made models. The Bewick, Fleischman, and Thirsk model illustrates the 2D progressive (2DP) and 2D instantaneous (2DI) deposition of Li_2S based on Equations (1) and (2) [1,2], respectively, while the Scharifker–Hills model depicts the 3D progressive (3DP) and 3D instantaneous (3DI) precipitation of Li_2S sequentially according to Equations (3) and (4) [3,4]. The parameters j and j_m are, respectively, assigned to the current responses at the time of t and t_m in Fig. S3.

$$\frac{j}{j_m} = \left(\frac{t}{t_m}\right)^2 \left\{exp\left[\frac{-2\left(t^3 - t_m^3\right)}{3t_m^3}\right]\right\} \quad (1)$$

$$\frac{j}{j_m} = \left(\frac{t}{t_m}\right) \left\{exp\left[\frac{t^2 - t_m^2}{2t_m^2}\right]\right\} \quad (2)$$

$$\frac{j}{j_m} = \left(\frac{1.2254}{\frac{t}{t_m}}\right)^{0.5} \left\{1 - exp\left[2.3367\left(\frac{t}{t_m}\right)^2\right]\right\} \quad (3)$$

$$\frac{j}{j_m} = \left(\frac{1.9542}{\frac{t}{t_m}}\right)^{0.5} \left\{1 - exp\left[1.2564\left(\frac{t}{t_m}\right)\right]\right\} \quad (4)$$

DFT calculation

We employed first-principles to perform density functional theory (DFT) calculations within the generalized gradient approximation (GGA) using the Perdew-Burke-Ernzerhof (PBE) formulation [5-7]. We chose the projected augmented

wave (PAW) potentials to describe the ionic cores and take valence electrons into account using a plane wave basis set with a kinetic energy cutoff of 520 eV. The GGA+U method was adopted in our calculations [8,9]. The value of the effective Hubbard U was set as 4.729 for Fe atoms. Partial occupancies of the Kohn−Sham orbitals were allowed using the Gaussian smearing method with a width of 0.05 eV. The electronic energy was considered self-consistent when the energy change was smaller than 10^{-5} eV. A geometry optimization was considered convergent when the energy change was smaller than 0.05 eV $Å^{-1}$. The Brillouin zone integration was performed using 3×3×1 Monkhorst-Pack k-point sampling for a structure. In addition, the Spin had been considered with ISPIN=2 and an initial magnetic moment. The crystal plane (104) of α-Fe_2O_3, which exhibits the strongest diffraction intensity of the XRD pattern, was selected for the calculations.

We have chosen the projected augmented wave (PAW) potentials to describe the ionic cores and take valence electrons into account using a plane wave basis set with a kinetic energy cutoff of 450 eV to calculate the Gibbs free energy. The electronic energy was considered self-consistent when the energy change was smaller than 10^{-5} eV. A geometry optimization was considered convergent when the force change was smaller than 0.04 eV/Å. The vacuum spacing in a direction perpendicular to the plane of the structure is 20 Å. The van der Waals interactions were considered by the method of the Grimme (DFT+D3) [10]. The Brillouin-zone integration was sampled with a Monkhorst-Pack mesh of 2×2 ×1 in the structural relaxation calculations. Spin polarization method was adopted to describe magnetism of slab models. The minimum energy paths (transition states, TS) were conducted using climbing nudged elastic band (CI-NEB) method [11].

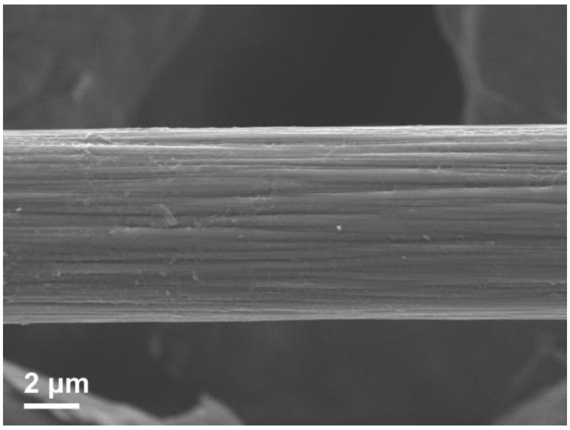

Figure S1. SEM image of the blank CP.

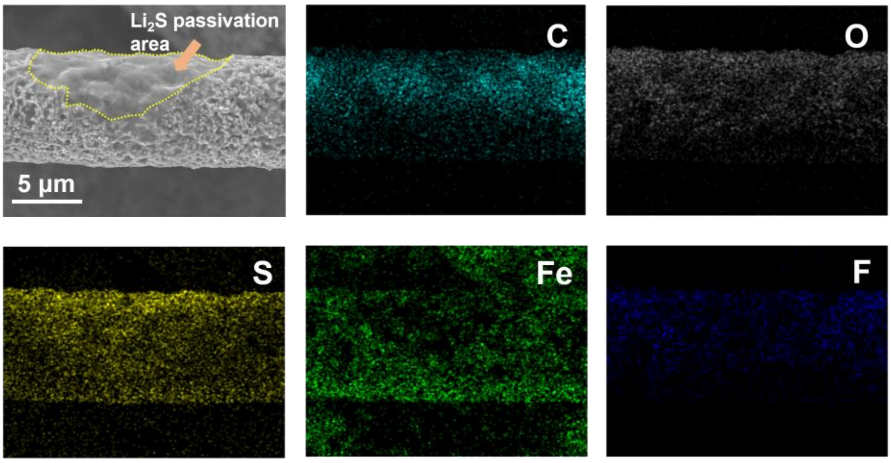

Figure S2. EDS mappings of α-Fe_2O_3/CP after Li_2S deposition with 0 mT MF.

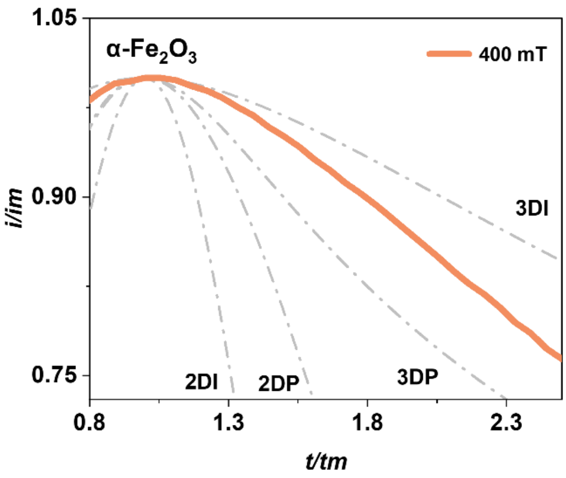

Figure S3. Li_2S nucleation models of α-Fe_2O_3 with 400 mT MF.

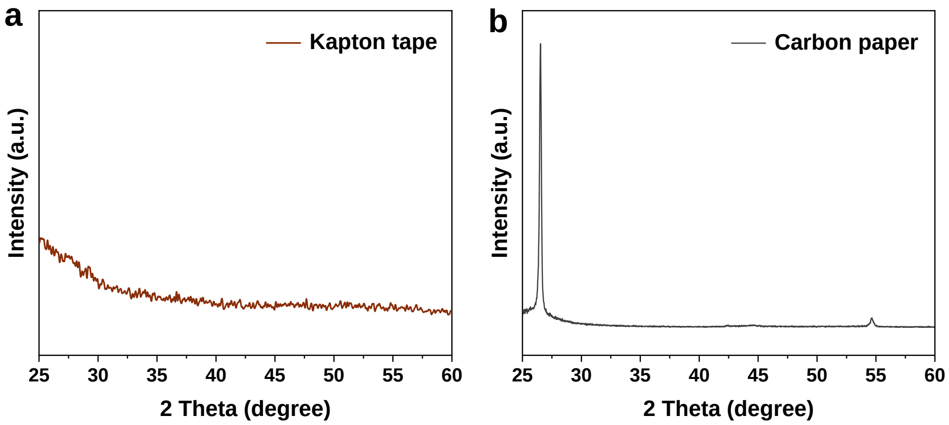

Figure S4. XRD patterns of the Kapton tape with a thickness of 10 μm (a) and carbon paper (b).

Note: Due to the overlap between the (111) peak of Li_2S and the strong diffraction peak of carbon paper at ~26°, the (220) secondary peak of Li_2S was selected for the FWHM analysis.

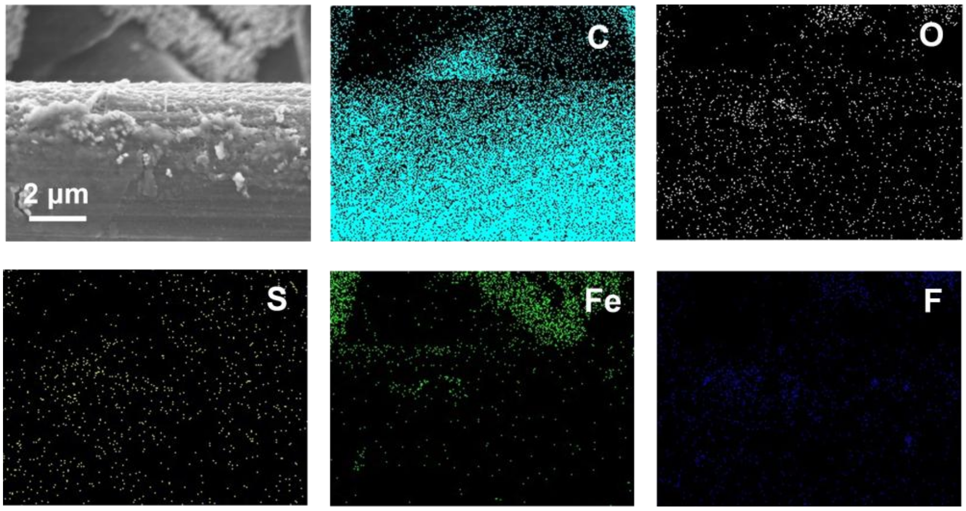

Figure S5. EDS mappings of α-Fe_2O_3/CP after Li_2S dissociation with 0 mT MF.

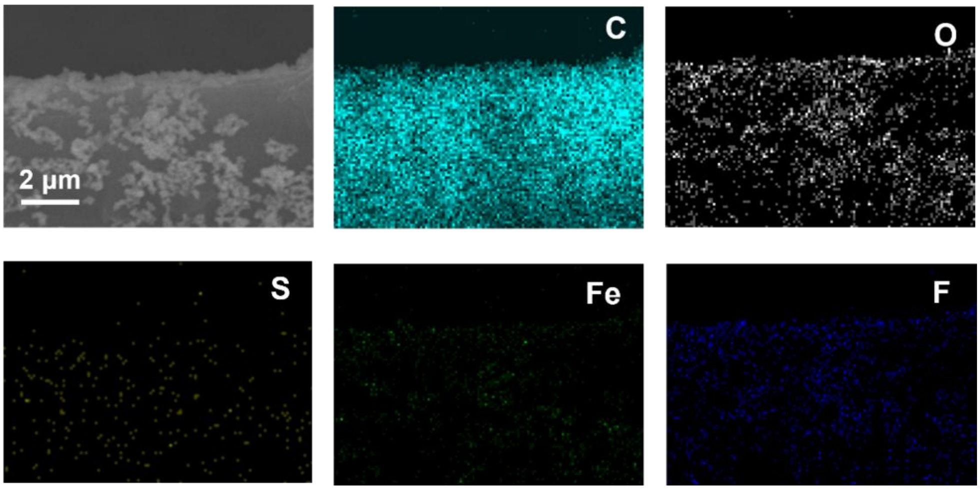

Figure S6. EDS mappings of α-Fe_2O_3/CP after Li_2S dissociation with 400 mT MF.

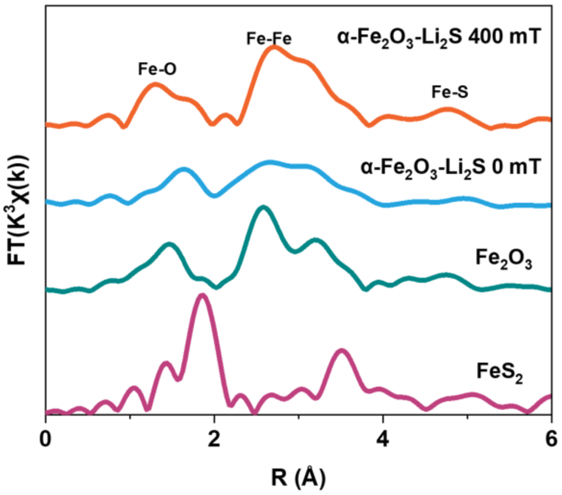

Figure S7. FT-EXAFS spectra of the FeS_2, α-Fe_2O_3, α-Fe_2O_3-Li_2S 0 mT, and α-Fe_2O_3-Li_2S 400 mT.

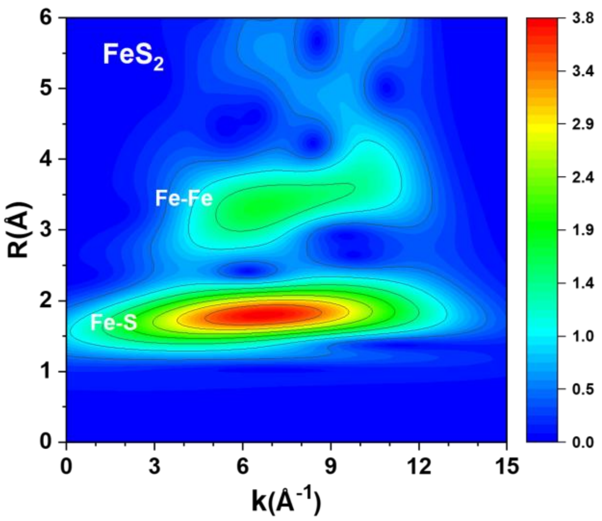

Figure S8. 2D WT-EXAFS for the k^3-weighted Fe K-edge of FeS_2.

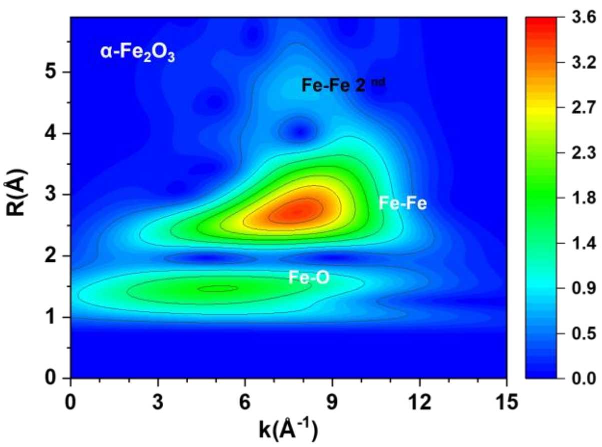

Figure S9. 2D WT-EXAFS for the k^3-weighted Fe K-edge signals of α-Fe_2O_3.

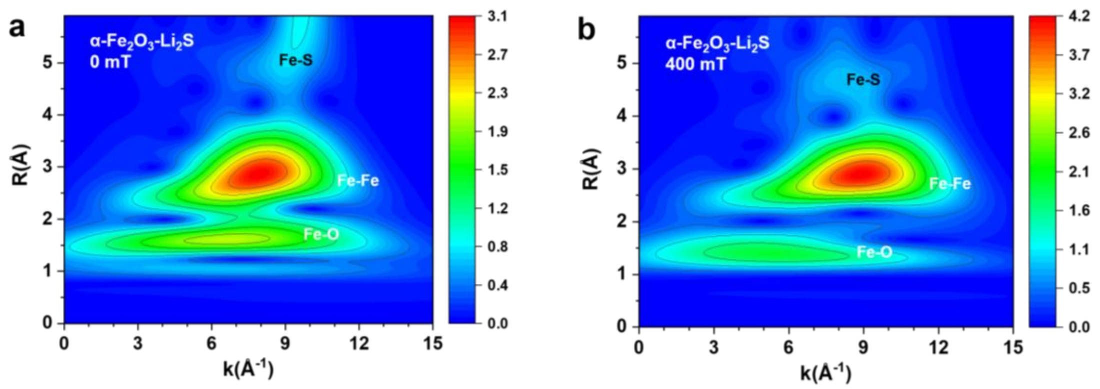

Figure S10. 2D WT-EXAFS for the k^3-weighted Fe K-edge signals of α-Fe_2O_3-Li_2S 0 mT (a) and α-Fe_2O_3-Li_2S 400 mT (b).

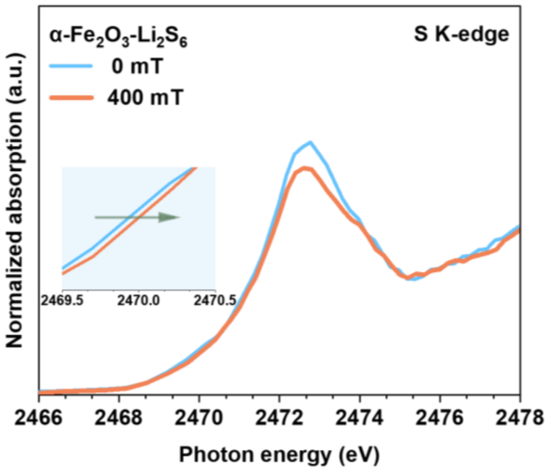

Figure S11. S K-edge XANES of α-Fe_2O_3-Li_2S_6 under 0 mT and 400 mT MF. The inset shows a magnified view in the energy range of 2469.5–2470.5 eV [12].

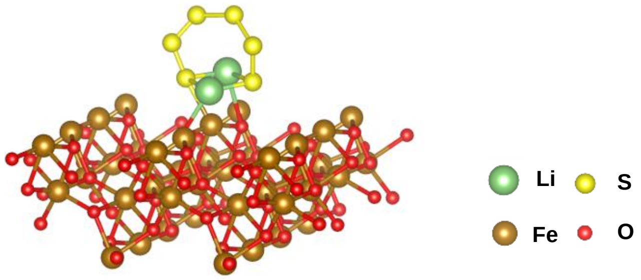

Figure S12. Absorption structure model of α-Fe_2O_3 (104)-Li_2S_6.

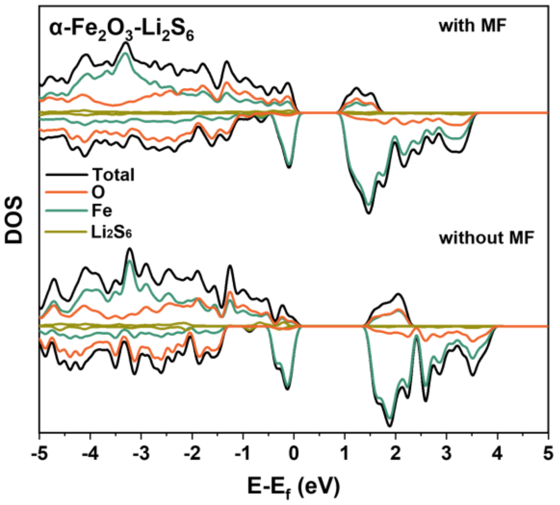

Figure S13. DOS of α-Fe_2O_3-Li_2S_6 with and without MF.

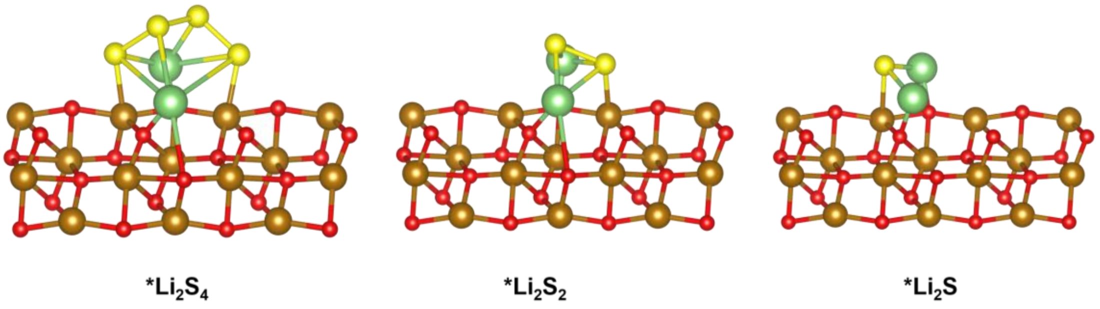

Figure S14. The optimized absorption configurations of discharging process from Li_2S_4 to Li_2S on α-Fe_2O_3 without MF.

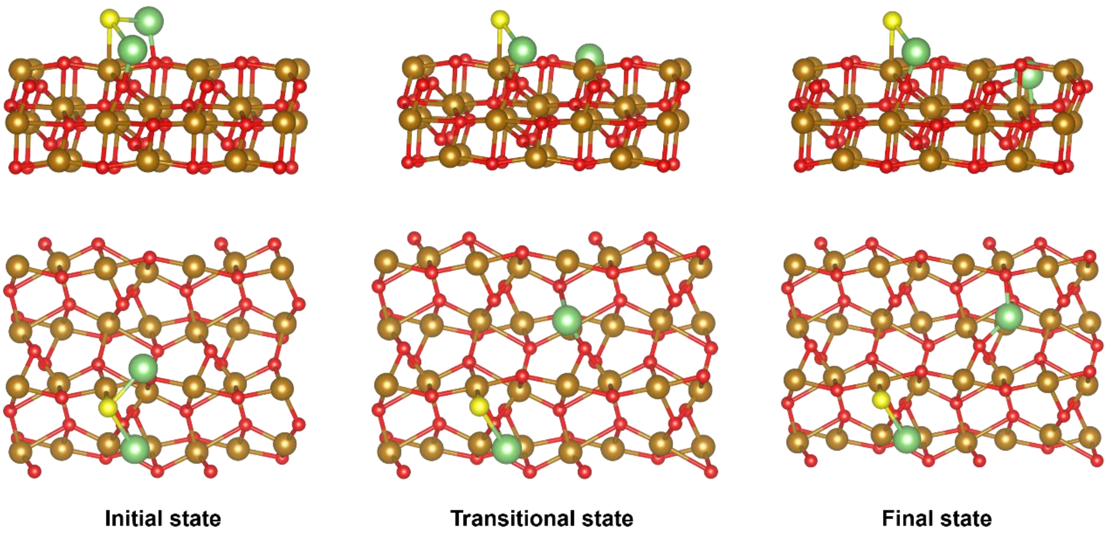

Figure S15. Absorption structure models of Li_2S dissociation on α-Fe_2O_3 (104) surface without MF.

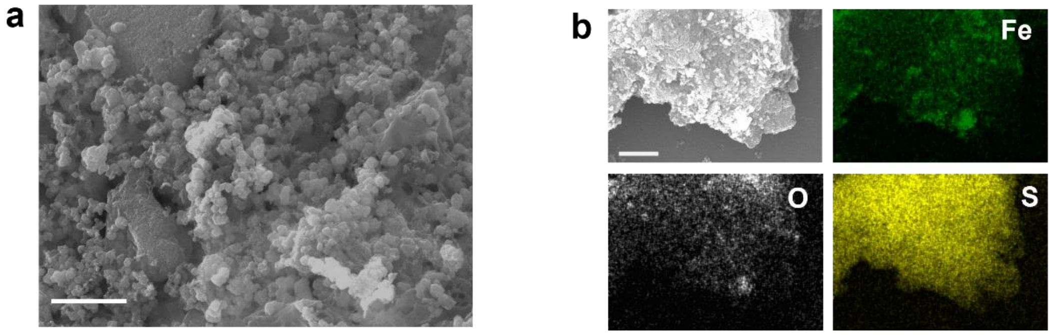

Figure S16. a, b) SEM image a) and EDS mappings b) of α-Fe_2O_3/S. The scale bars are 2 μm.

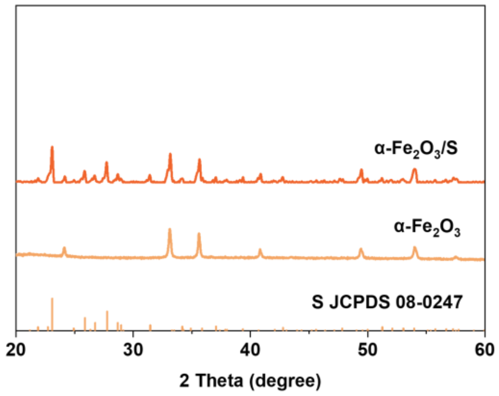

Figure S17. XRD patterns of α-Fe_2O_3, α-Fe_2O_3/S and standard card of S (JCPDS 08-0247).

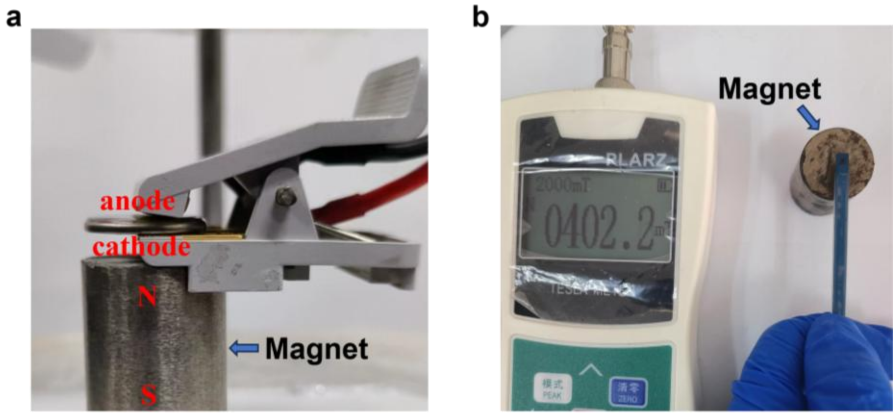

Figure S18. a) Optical photo of a coin battery under 400 mT MF. b) Measurement of magnetic strength at a height of 5 mm above the surface of the magnet, using a hand-held Tesla meter. The magnetic strength is 402.2 mT, approximately equal to 400 mT.

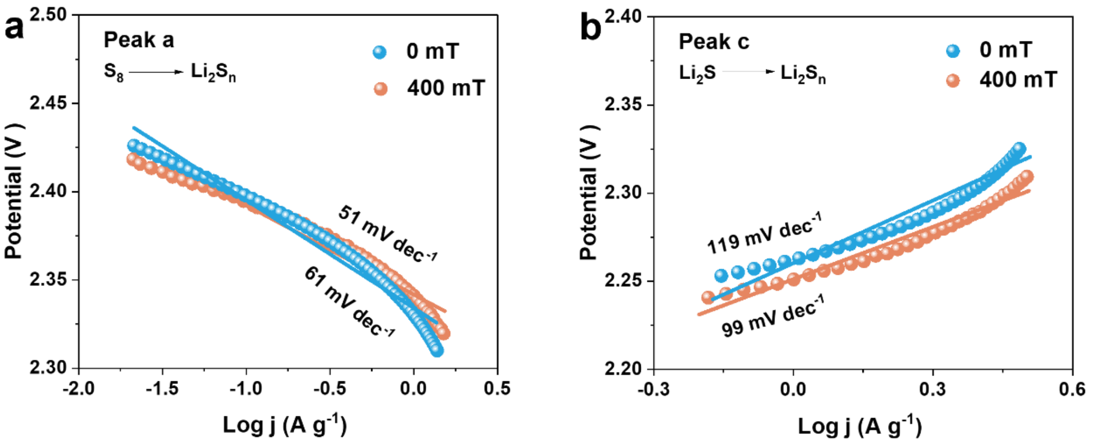

Figure S19. a, b) Tafel plots of peaks a and c on CV curves of α-Fe_2O_3/S with 0 mT and 400 mT MF.

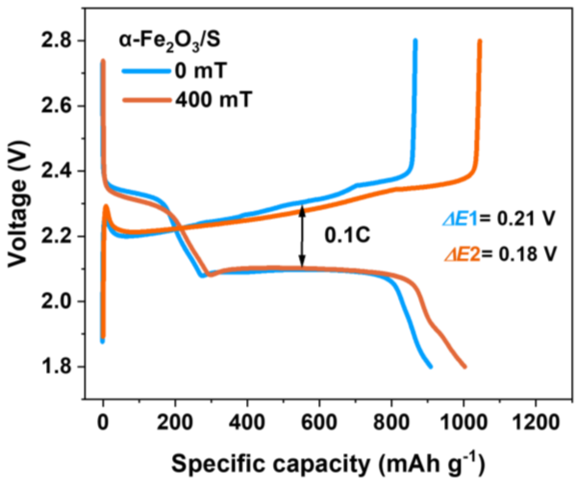

Figure S20. The charge/discharge curves of α-Fe_2O_3/ S under 0 mT and 400 mT MF.

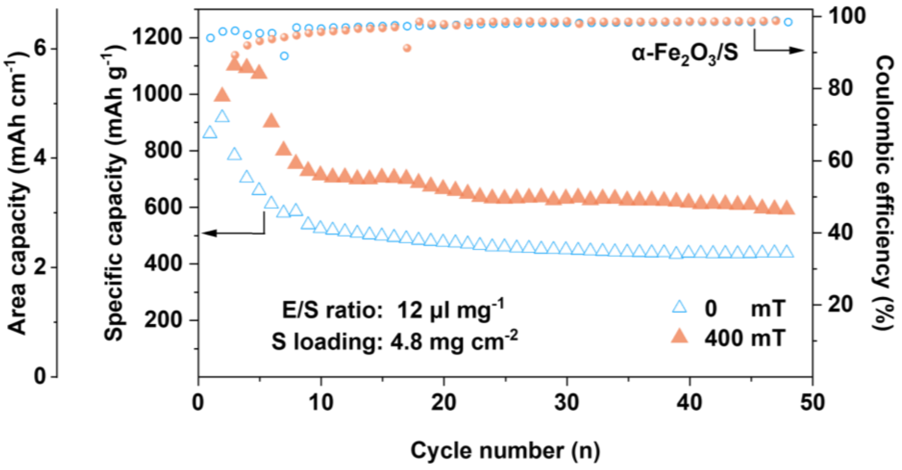

Figure S21. Cycling performances of α-Fe_2O_3/ S cathodes with high sulfur loading of 4.8 mg cm^{-2} at 0.05 C.

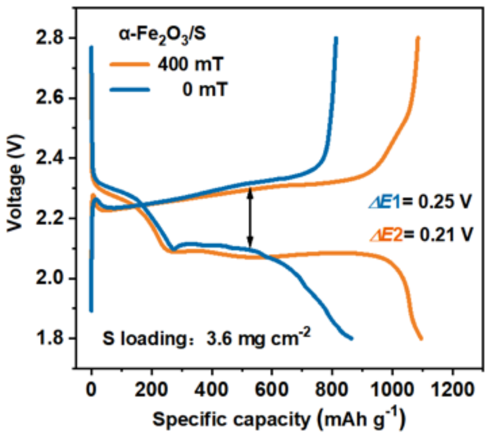

Figure S22. Charge/discharge curves of α-Fe_2O_3/S electrodes at 0.05 C under 0 mT and 400 mT MF.

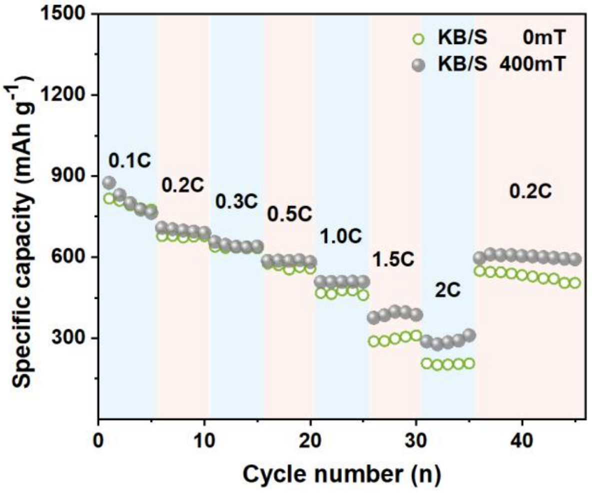

Figure S23. Rate performances of KB/S under 0 mT and 400 mT MF.

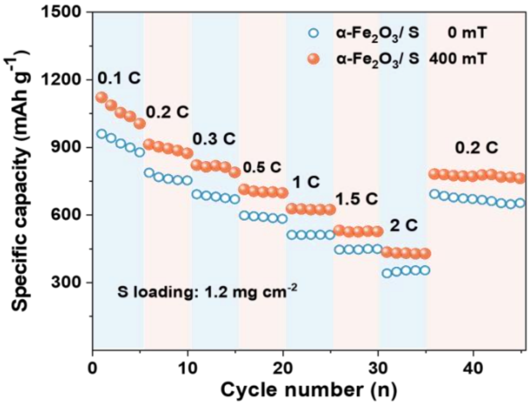

Figure S24. Rate performances of α-Fe_2O_3/S under 0 mT and 400 mT MF.

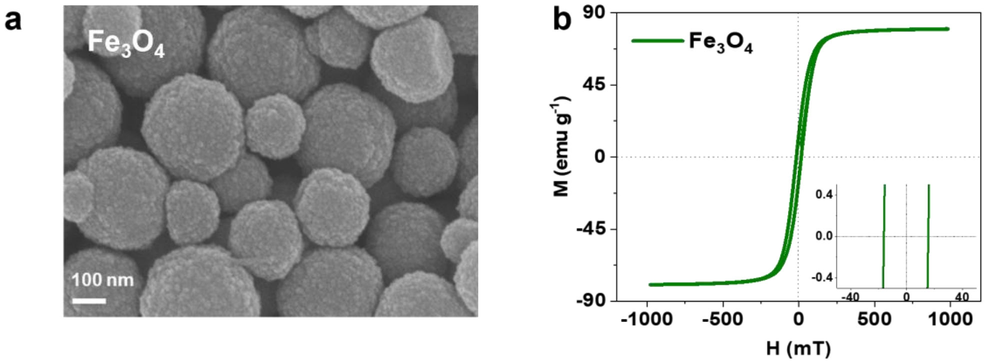

Figure S25. a) SEM image of Fe_3O_4 nanoparticles, the scale bar is 100 nm. b) Magnetic hysteresis loop of Fe_3O_4.

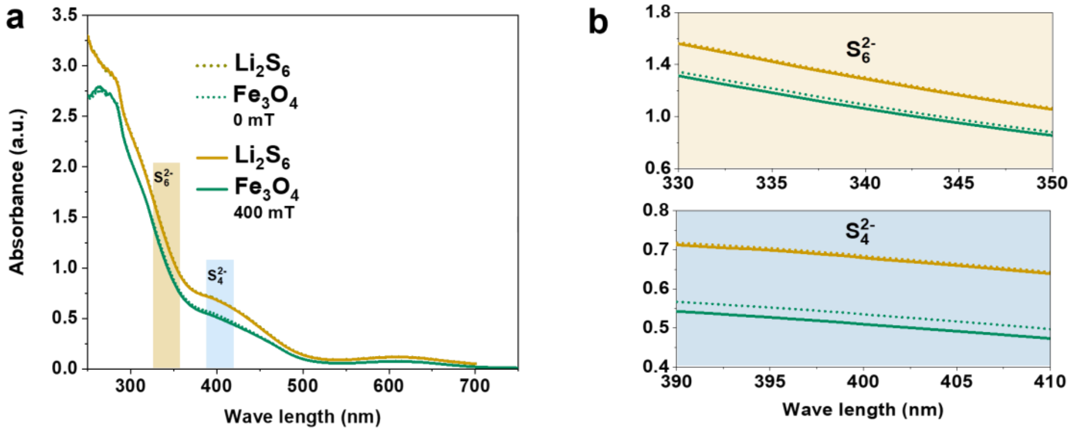

Figure S26. a) UV-vis absorption spectra of the Li_2S_6 solution with Fe_3O_4 after resting 2 h with and without 400 mT MF. b) Enlarged view of absorbance curves in the ranges of 330-350 nm and 390-410 nm.

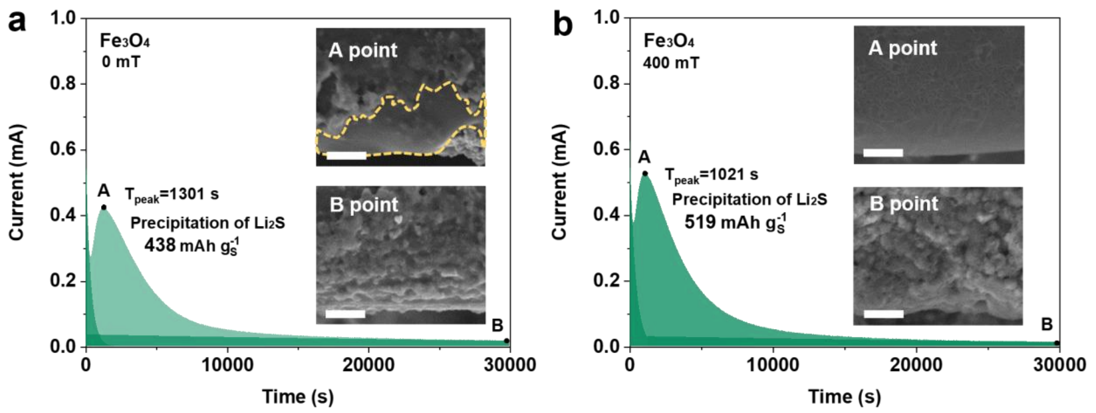

Figure S27. a, b) Li_2S deposition curves of Fe_3O_4 with 0 mT and 400 mT MF. The SEM images of Li_2S deposition on CP at points A, B and points A', B' of the deposition curves, the scale bar is 2 μm.

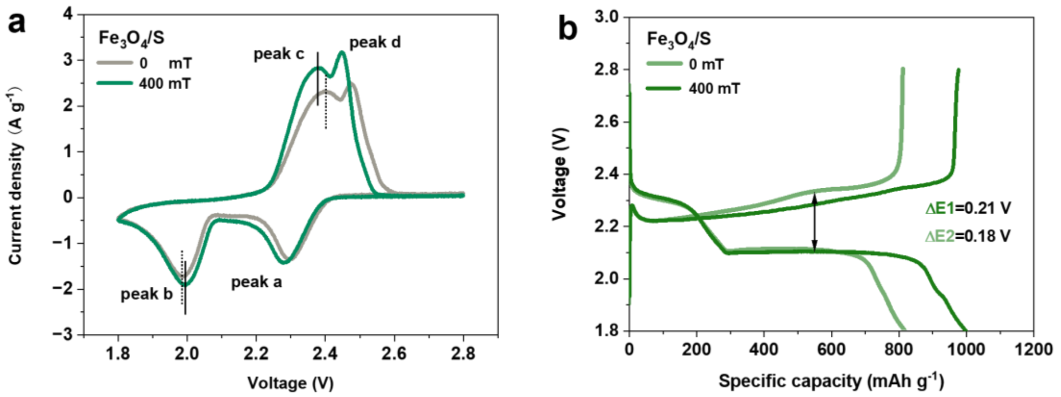

Figure S28. a) CV curves of Fe_3O_4/S with 0 mT and 400 mT MF. b) Charge/discharge curves of Fe_3O_4/S with 0 mT and 400 mT MF at 0.1 C.

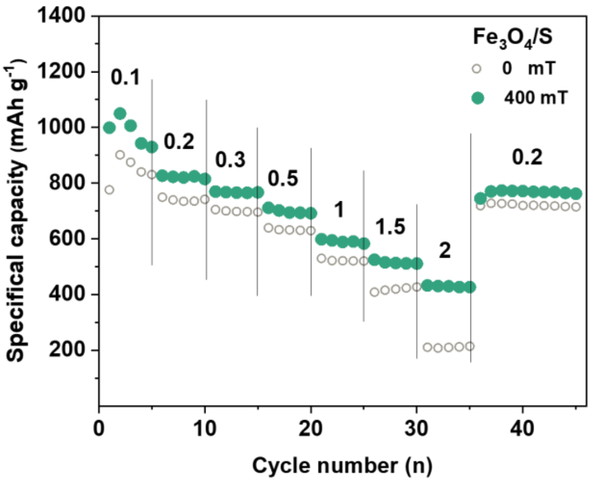

Figure S29. Rate performances of Fe_3O_4/S electrodes with 0 mT and 400 mT MF.

Table S1. Element contents on EDS mappings of Figure S5 and Figure S6.

**α-Fe_2O_3/CP**	**Element**	**Weight percent** (%)	**Atomic percent** (%)
**0 mT**	C	71.93	84.51
	O	10.68	9.43
	F	2.62	1.94
	S	2.08	0.91
	Fe	12.69	3.21
	Total		100
**400 mT**	C	73.62	84.96
	O	12.16	10.52
	F	2.74	1.80
	S	0.28	0.11
	Fe	11.20	2.61
	Total		100

Table S2. Fe K-edge EXAFS curves fitting parameters of α-Fe_2O_3-Li_2S 400, 0 mT, and pristine α-Fe_2O_3.

**α-Fe_2O_3-Li_2S**	**Path** (Fe center)	$\boldsymbol{N}$	$\boldsymbol{R}$(Å)	$\boldsymbol{\sigma^2}$(Å^2)	$\boldsymbol{\Delta E_0}$ (eV)	**R factor**
**400 mT**	Fe-O	5.1	1.97	0.0136	-4.786	0.013
	Fe-Fe	2.4	3.15	0.004		
	Fe-O	1.9	3.82	0.0086		
	Fe-S	11.5	5.27	0.0041		
**0 mT**	Fe-O	2.1	2.03	0.0033	4.983	0.019
	Fe-Fe	2.6	2.96	0.0034		
	Fe-O	1.2	3.72	0.0139		
	Fe-S	3.3	5.65	0.0111		
**pristine α-Fe_2O_3**	Fe-O	4.1	1.97	0.0130	0.961	0.009
	Fe-Fe	4.3	3.02	0.0204		
	Fe-O	1.2	3.58	0.0862		

N, coordination number; *R*, distance between absorber and backscatter atoms; σ^2, Debye-Waller factor to account for both thermal and structural disorders; ΔE_0, inner potential correction; *R* factor indicates the goodness of the fit. The amplitude reduction factor (S_0) was fixed at 1. Fitting range: Δk= 1.5~10 0 mT, 3~11.3 Å^{-1} 400 mT, ΔR= 1~5.2 Å.

Table S3: Performance comparison of the high loading Li-S pouch cells between this work and recent reports.

Materials	Areal S loading (mg cm^{-2})	Initial capacity	Capacity retention	Cycle number	E/S ratio (μL mg^{-1})	Ref.
**α-Fe_2O_3/S (S 60 wt %)**	**3.6**	**1150 mAh g^{-1} (0.05 C)**	**67.1%**	**30**	**7**	**This work**
ZnS@HPCS	4.6	1190.2 mAh g^{-1} (0.1 C)	70.3%	40	10	13
Al_2O_3@mG	2.2	5.4 mAh cm^{-2} (0.2 C)	73.1%	70	4.8	14
Co/MoN	2.0	988 mAh g^{-1} (0.2 C)	61.2%	20	10	15
SNGO/Li	2.5	924 mAh g^{-1} (0.2 C)	80%	100	3.3	16
S-N/CF@V_2CT_x	6.0	1667 mAh g^{-1} (0.1 C)	52.5%	20	3	17
S@ZnN_4-NC	2.3	953 mAh g^{-1} (0.1 C)	79.1%	100	4.2	18
TiO_2/TiN	3.7	928.5 mAh g^{-1} (0.1 C)	69.3%	55	6.5	19

Table S4. Comparing the performances of electrodes with/without catalysts under 0 mT and 400 mT MF from Figure S24 and Figure S25.

**Current density (A g^{-1})**	**Capacity (mAh g^{-1})**				**Capacity retention (%)**			
	**α-Fe_2O_3/S (0 mT)**	**KB/S (0 mT)**	**α-Fe_2O_3 /S (400 mT)**	**KB/S (400 mT)**	**α-Fe_2O_3/S (0 mT)**	**KB/S (0 mT)**	**α-Fe_2O_3 /S (400 mT)**	**KB/S (400 mT)**
0.1 C	957.8	817.1	1085.1	875.2	/	/	/	/
0.2 C	765.8	672.9	900.2	708.5	79.9	82.2	**82.9**	80.9
0.3 C	689.3	637.1	809.1	656.6	72.0	77.9	**75.5**	75.0
0.5 C	595.1	557.7	711.1	586.7	62.1	68.2	**65.5**	67.0
1 C	508.8	468.1	623.4	508.8	53.1	57.2	**57.5**	58.1
1.5 C	443.0	328.8	522.1	375.9	46.2	40.2	**48.1**	43.0
2 C	337.7	237.4	432.1	288	35.3	29.1	**39.8**	32.9
0.2 C	650.5	528.8	759.7	592.1	67.9	64.7	**70.0**	67.6

References

1. Paunovic M *et al*. *Fundamentals of electrochemical deposition*. (*John Wiley & Sons*, Inc., 2006).
2. Bewick A *et al.* Kinetics of the electrocrystallization of thin films of calomel. *Trans Faraday Soc* 1962; **58**: 2200-16.
3. Scharifker B *et al*. Theoretical and experimental studies of multiple nucleation. *Electrochim Acta* 1983; **28**: 879-89.
4. Scharifker B *et al.* Electrocrystallization of copper sulphide (Cu_2S) on copper. *Electrochim Acta* 1984; **29**: 261-66.
5. Kresse G *et al*. Efficiency of ab-initio total energy calculations for metals and semiconductors using a plane-wave basis set. *Comput Mater Sci* 1996; **6**: 15-50.
6. Kresse G *et al*. Efficient iterative schemes for ab initio total-energy calculations using a plane-wave basis set. *Phys Rev B* 1996; **54**: 1116-86.
7. Perdew J P *et al*. Generalized gradient approximation made simple. *Phys Rev Lett* 1996; **77**: 3865-68.
8. Kresse G *et al*. From ultrasoft pseudopotentials to the projector augmented-wave method. *Phys Rev B* 1999; **59**: 1758-75.
9. Blöchl P E *et al*. Projector augmented-wave method. *Phys Rev B* 1994; **50**: 17953-79.
10. Grimme S *et al*. A consistent and accurate ab initio parametrization of density functional dispersion correction (DFT-D) for the 94 elements H-Pu. *J Chem Phys* 2010; **132**: 154104.
11. Henkelman G *et al*. A climbing image nudged elastic band method for finding saddle points and minimum energy paths. *J Chem Phys* 2000; **113**: 9901-04.
12. Cuisinier M *et al*. Radical or not radical: revisiting lithium–sulfur electrochemistry in nonaqueous electrolytes. *Adv Energy Mater* 2015; **5**: 1401801.
13. Ding H *et al*. Regulating Li_2S deposition and accelerating conversion kinetics through intracavity ZnS toward low-temperature lithium–sulfur batteries. *Nano Lett* 2024; **24**:15118-26.
14. Gu J *et al*. Sustaining vacancy catalysis via conformal graphene overlays boosts practical Li–S batteries. *Energy Environ Sci* 2025; **18**: 5940-5951.
15. Kong Y *et al*. Co/MoN invigorated bilateral kinetics modulation for advanced lithium–sulfur batteries. *Adv Mater* 2024; **36**: 2310143.
16. Ni S *et al*. A 3D framework with Li_3N–Li_2S solid electrolyte interphase and fast ion transfer channels for a stabilized lithium-metal anode. *Adv Mater* 2023; **35**: 2209028.

17. Jin Q *et al*. Nanofiber-interlocked V_2CTx hosts enriched with 3D lithiophilic and sulfophilic sites for long-life and high-rate lithium–sulfur batteries. *Adv Funct Mater* 2024; **34**: 2309624.
18. Zhang X *et al*. Single zinc atom aggregates: synergetic interaction to boost fast polysulfide conversion in lithium-sulfur batteries. *Adv Mater* 2023; **35**: 2208470.
19. Yang S *et al*. In situ constructing a TiO_2/TiN heterostructure modified carbon interlayer for balancing the surface adsorption and conversion of polysulfides in Li–S batteries. *Adv Energy Mater* 2024; **14**: 2400648.
